# Clinical aspects of umbilical cord cannulation during transfer from the uterus to a liquid-based perinatal life support system for extremely premature infants a qualitative generic study

**DOI:** 10.1371/journal.pone.0290659

**Published:** 2023-12-21

**Authors:** M. Verrips, J. S van Haren, S. G Oei, A Moser, M. B. Van der Hout-van der Jagt

**Affiliations:** 1 Department of Obstetrics and Gynecology, Máxima Medical Center, Veldhoven, the Netherlands; 2 Faculty of Industrial Design, Eindhoven University of Technology, Eindhoven, the Netherlands; 3 Department of Family Practice, Maastricht University, Maastricht, the Netherlands; 4 Faculty of Electrical Engineering, Eindhoven University of Technology, Eindhoven, the Netherlands; Ankara Etlik City Hospital, TURKEY

## Abstract

A liquid-based perinatal life support system (PLS) for extremely premature infants (born before 28 week of gestational age) envisions a connection between the infant’s native umbilical cord and an artificial placenta system through cannulation. This system mimics a natural mothers’ womb to achieve better organ maturations. The objective of this study is to gain insight into the clinical focus points of umbilical cord cannulation and how cannulation should be addressed in extremely premature infants during the transfer from the uterus to an in-utero simulating liquid-based PLS system. We performed an explorative qualitative study. Twelve medical specialists with knowledge of vessel cannulation participated. We collected data through twelve interviews and two focus group discussions. Data were analyzed using inductive content and constant comparison analysis via open and axial coding. Results were derived on the following topics: (1) cannulation technique, (2) cannula fixation, (3) local and systemic anticoagulation, and (4) vasospasm. A side-entry technique is preferred as this may decrease wall damage, stabilizes the vessel better and ensures continuous blood flow. Sutures, especially via an automatic microsurgery instrument, are favored above glue, stents, or balloons as these may be firmer and faster. Medication possibilities for both vasospasm and anticoagulation should function locally since there were uncertainties regarding the systemic effects. According to the findings of this research, the needed umbilical cord cannulation method should include minimal wall damage, improved vascular stability, blood flow maintenance, a strong fixation connection, and local anticoagulation effect.

## Introduction

According to the World Health Organization, world-wide approximately 15 million neonates are born prematurely, annually [1]. To provide an alternative to neonatal intensive care for extreme premature infants, the concept of the artificial amnion and placenta technology (AAPT) is investigated, to postpone the transition of fetal to neonatal physiology after preterm birth [2]. In this technology, the extremely premature infant is incubated in a system that is connected to an artificial placenta and mimics the in-utero environment. To realize this connection, circulatory access is required to maintain fetal physiology and secure oxygenation. Umbilical cord cannulation is commonly performed in premature infants to directly access the vascular system. It is a safe route for intravenous delivery of nutrients, medication and fluid [3]. The umbilical cord consists of two umbilical arteries and one umbilical vein that form a helix, embedded in Wharton’s jelly. Wharton’s jelly acts as connective tissue that protects the vessels from rupturing [4]. Depending on the gestational age of the infant, the cord length ranges from 30 to 100 cm, with a diameter from around 3.2 mm to 16.7 mm [4], and difference in Wharton’s jelly thickness.

Previous studies on AAPT have been conducted in animal experiments [5–10], but not in humans. So far, little research has been published on umbilical cord vessel cannulation in human premature infants when being transferred from the maternal womb to an AAPT system to continue fetal organ maturation and growth [5, 9, 11]. The purpose of this study was to gain clinical insight into the focus points of umbilical cord cannulation and how cannulation should be addressed in (extremely) premature infants during the transfer from maternal womb to an extra-corporal, in-utero stimulating system, known as perinatal life support (PLS) system.

## Methods

**Fig 1:**
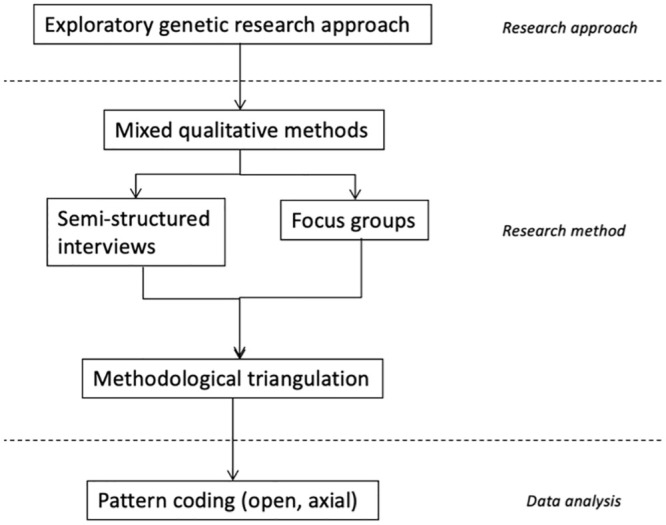
Research process overview.

An exploratory qualitative study was conducted from November 2021 until May 2022 to collect clinical knowledge regarding vessel (cannulation) (Fig 1). In this study, semi-structured interviews and focus groups were organized, as both in-dept knowledge and consensus was required. Medical specialists with knowledge regarding vessel cannulation were contacted (inclusion criteria). No exclusion criteria were set. Since medical experts are often well-connected to related experts on affiliated topics, snowball sampling was used to recruit additional participants. The first participants were recruited among national specialists affiliated with the PLS consortium and secondly, through snowball sampling other national and international professionals were approached and contacted. Thereafter, focus groups were organized to discuss gathered information regarding umbilical cord cannulation. In addition, all specialists that were interviewed were also asked to participate in the focus groups. Participants could join the interview and focus group discussions either in person or through videoconferencing [MS Teams, version 1.4.00.22472, Microsoft, New York, America]. Both focus groups took place digitally via Microsoft Teams. This hybrid format was necessary due to COVID-19 regulations and workplace localization across the Netherlands.

The Daily Board of the Medical Ethics Committee Máxima MC stated that the rules laid down in the Medical Research Involving Human Subjects Act do not apply to this research.

A total number of twelve medical specialists participated in the interviews, these are referred to as I1-I12, respectively (Table 1). Five of them also participated in one or both of focus groups, referred to as F1 and F2, respectively (four responders in the first and three responders in the second focus group). The interviewees consisted of three gynecologists, two neonatologists, two vascular surgeons, one plastic surgeon, one intervention radiologist, one pediatric surgeon, and one internist-hematologist. They all worked in either university hospitals or teaching hospitals. The interviews lasted between 15–85 minutes with an average of 45 minutes, and the focus groups about one hour. Verbal consent was provided at the beginning of each interview and focus group. All were recorded and transcribed verbatim by one of the authors (MV).

**Table 1:** List of interviews.

Interviewee	Abbreviations	Specialism	Interview date	Interview duration	Placement
1	I1	Intervention radiology	8-2-2022	30 minutes	Physical
2	I2	Plastic surgery	1-12-2021	30 minutes	Physical
3	I3	Gynaecology	29-10-2021	1h 20 minutes	Digital
4	I4	Vascular surgery	11-3-2022	45 minutes	Digital
5	I5	Neonatology	12-11-2021	53 minutes	Digital
6	I6	Internal hematology	18-3-2022	41 minutes	Digital
7	I7	Pediatric surgery	1-12-2021	48 minutes	Digital
8	I8	Gynaecology	26-11-2021	35 minutes	Digital
9	I9	Gynaecology	9-11-2021	15 minutes	Physical
10	I10	Neonatology	24-2-2022	42 minutes	Digital
11	I11	Vascular surgery	4-11-2021	30 minutes	Physical
12	I12	Neonatology	12-11-2021	53 minutes	Digital
1	F1	Gynaecology, neonatology, vascular surgeon	17-2-2022	1h 5 minutes	Digital
2	F2	Gynaecology, neonatology, vascular surgeon	3-3-2022	58 minutes	Digital

A semi-structured interview guide, based on desk research of literature, was prepared for the interviews (Table 2). Similar questions were further discussed in the focus groups. The interview questions focused on various steps and aspects of the cord cannulation procedure [12]. Depending on specific knowledge of the experts, more in-depth questions were asked in the interviews, to gain more insight in the subtopics. Next to the interviews, the focus groups were aimed to get in-depth insights about topics mentioned during the interviews and to facilitate discussion on certain ideas that were brought up during the interviews to come up with consensus, guided by PowerPoint Microsoft.

**Table 2:** Semi-structured interview guideline.

Clarification & ethical questions	
1.	Do I have permission to record this interview?
2.	Could you introduce yourself: background and current function?
Questions	
1.	How do you envision the umbilical cord cannulation in (extreme) premature infants?
2.	What methods/techniques can be used to puncture the umbilical cord? And what is the best way to do this?
3.	How can we fixate the inserted cannula to the umbilical cord?
4.	What kind of cannula should be used, when referring to material and length, for example?
5.	How should vasospasm of the umbilical cord be handled?
6.	How should coagulation of the umbilical cord be handled?
7.	What is the time window for umbilical cord cannulation? In how much time should the cord be cannulated?
8.	Who will perform the cannulation?
9.	What is your current experience with cannulating vessels?
10.	How can different vessel of the umbilical cord be identified?

### Data analysis

The transcripts were transcribed and coded using qualitative analysis software (Atlast.ti, *22*.*0*.*1*, *Scientific Software Development*, *2022*, *Berlin*). Inductive content analysis, including open and axial coding, was conducted to minimize preconceived notions and biases, and to crosslink information collected in the semi-structured interviews and focus groups [13]. The subcategories derived from interviews or focus group were constantly compared to formulate categories. Thematic saturation occurred after the second focus group, as no new categories emerged from the data [14].

### Methodological considerations

To ensure internal validity, data and source triangulation and member checking occurred [15]. Data triangulation was performed through multiple methods, such as interviews and focus groups. Source triangulation was secured by including specialists with different backgrounds and sources of experience. The interviewees were contacted for respondent validation (member checking) and asked to review the transcripts. External validity was ensured by providing a clear description of the research and through recording, transcribing, and coding the results. The results were then sent to the interviewees for validation. It concerns the aspect of applicability, as to provide transparency on its methods and decisions [16].

## Results

In this study four main results emerged on umbilical cord cannulation during the transfer to the PLS system: cannulation technique, cannula fixation, anticoagulation, and vasospasm. Illustrative verbatim comments per category are shown in Box 1 of S3 File.

### Cannulation technique

In the interviews three main cannulation techniques are mentioned: transversal dissection, Seldinger-like technique, and side-entry technique. *Transversal dissection* is described as a complete dissection of the umbilical cord, eventually showing three transversal vessels [17]. Several participants mentioned that transversal dissection could be beneficial for artery and vein identification [I3, I12, F1]. Moreover, in case of a prior unusable transversal cord surface, it is relatively easy to achieve a new transversal dissection, closer to the abdominal wall [I12]. Furthermore, several challenges were mentioned, including blood leakage from the cord that impairs vision, intima withdrawal, vasospasm, and blood flow interruption in which the fetus does not get oxygen [I3, I5, I7, I10, I11, F1 and F2].

With the *Seldinger-like technique*, a blood vessel is cannulated via a side-puncture with the use of a needle [18]. I10-12 describe this technique as an easier and faster route in which the blood flow continues, and less intima withdrawal occurs compared to the transversal dissection (Quote 1, Box 1 in S3 File). If this technique fails, transversal dissection may still be a possibility. I3, I10, I11, I12 have also indicated that this technique may require more time and could cause wall damage; they are concerned about the small diameter of the vessels [F1].

The last technique that was proposed was the *side-entry technique*. This side-entry technique consists of a longitudinal incision in the umbilical cord vessels in which the cannula is placed directly, and does not require a needle or guidewire [19]. It is not widely used [I10], as only few clinics have experience with it. It was thought to be faster, easier, and better in stabilizing the vessels [I3, I10, I11]. Moreover, no inner wall withdrawal and/or damage is related to this technique [I10]. Consensus on its potential was achieved in focus group 1.

### Cannula fixation

Duration as well as steadiness and firmness of the cannula is important for cannula fixation (Quote 2, Box 1 in S3 File). Concerning the duration of fixation, glue is mentioned as fixation method since it is assumed to only take seconds to be performed [I3]. However, concerns regarding its solidity were expressed as it might be too fragile to counteract the blood flow pressure from within the vessel [I2, F1, F2]. It is referred to as a temporary solution [I11]. Moreover, the density/consistency may vary in a dry or wet environment (humidity) and concerns were made regarding its fetotoxicity [I8].

Expendable balloons and stents were also considered since both can be cannulated in a folded state and deflated in seconds [I3, I11]. Concerns were expressed regarding vessel damage when balloons and stents are used [I4, I11, F1, F2]. To achieve faster and more specific cannulation in vessels with a small diameter, I2 suggested to look into the Coupler system, which is currently used for microsurgery and typically creates vessel anastomoses within minutes [20, 21].

During standard neonatal umbilical catheterization, sutures are placed in Wharton’s jelly, however, this tissue is very fragile and could rupture or break easily (Quote 3, Box 1 in S3 File).

### Anticoagulation

Intravenous lines increase the risk of thrombosis and clotting and could cause ischemia [22]. On the contrary, any anticoagulation products inserted in the neonate could also result in intracranial bleeding (Quote 4, Box 1 in S3 File). Both interviewed vascular surgeons use systemic anticoagulation in adults, whereas the neonatologists are more careful using systemic components in neonates, due to its increased risk of intracranial hemorrhage (Quote 5, Box 1 in S3 File).

Systemic anticoagulation can prevent thrombosis or ischemia in premature infants who had foreign material being introduced [23]. One such systemic anticoagulant is heparin. The interviewees suggested that depending on the hospital, a low dose of continuous intravenous heparin is advised in premature infants with an umbilical catheter [I5, I12]. Yet almost all interviewees emphasize to be aware of the use of heparin [F1]. The internist-hematologist suggests monitoring heparin based on clotting factors and time, for example by using anti-Xa blood levels, as it is used in adults. The downside of this coagulation factor is that the given value refers to a concentration range and is therefore not very specific [I4, I11, F1, F2]. That can be problematic in extreme premature infants since a slight change in intravenous concentration may cause hemorrhage as premature infants have an impaired clotting time [I4, I5]. Systemic administration of heparin based on its clotting time is therefore not preferred.

Local anticoagulation has the preference over systemic anticoagulation by all interviewees as it has the advantage that it will probably not come systemic and therefore has a decreased risk regarding intracranial hemorrhage (Quote 6, Box 1 in S3 File). An example of this could be a heparin- or ion-based cannula coating. I6 expressed that chemical stability and defragmentation regarding any cannula-coating should be focused on in future AAPT research.

### Vasospasm

I3, I5, I11, I12 mention that cold temperature, touch, and fixation of the umbilical cord result in vasoconstriction of vessels in the umbilical cord, and thus make it difficult to enter the vessel during cannulation (Quote 7, Box 1 in S3 File). It was also indicated that the exact duration of umbilical cord vessel constriction is unknown [I3].

Various medication may be used as treatment, including lidocaine, papaverine and nitroglycerine [F1]. Several clinicians have mentioned that lidocaine drops can be used to decrease vasospasm or vasoconstriction [I2, I7, I12]. It works fast, has a short-term effect, and does not enter the system [I12]. However, it was also mentioned that the beneficial effect of lidocaine is based on expert opinion and that literature is inconclusive about the real effect of dilatation [I7, F1]. Papaverine was also mentioned as an option. Papaverine works around 30–40 seconds and is only temporarily present [I1]. However, its effects are not known in human neonates [I5].

During the focus groups a vascular surgeon mentioned the use of papaverine in shunt surgery and that in lamb research it is administered via the intravenous line [F1]. Three ideas, with unclear effects still, were brought up: (1) local spray, (2) injection in the Whartons jelly or (3) intravenous and bathing the umbilical cord in this solution [I11]. In addition to papaverine, nitroglycerine was suggested as vasodilator as obstetricians use this via a spray for uterus relaxation, and gastro-enterology colleagues apply it as cream to reduce anus vasospasm [I3, I11]. None of the interviewees have had experience with this medicine. Altogether, the interviewees prefer the use of rapid-release medication instead of a slow-release medication due to the time limitation of umbilical cord cannulation.

Cannula material and fixation duration were only covered minimally in interviews and not discussed in focus group [F1-2]. It can however interact with the discussed topics and therefore requires more research.

## Discussion

Previous animal research with umbilical cord cannulation in lambs has been performed successfully, yet information on the clinical key points in umbilical cord vessel cannulation is only described in few [7]. According to our findings four main categories: canula technique, canula fixation, anticoagulation and vasospasm, were seen as crucial in umbilical cord cannulation. It indicates that for the required umbilical cord cannulation technique, less wall damage, better vessel stabilization, maintaining blood flow, tight fixation connection and local anticoagulation effects should be preferred.

### Cannulation technique

Through the focus groups a consensus regarding a preferred technique was achieved, namely the *side-entry technique*. The results showed that only a few clinicians use this, as other techniques have been predominated in the teaching of umbilical cannulation. This cannulation technique is endorsed by two articles [19, 24]. Other articles on animated-based AAPT studies reported several other cannulation methods, with varying results. The technique of Partridge et al. and Hornick et al. resulted in direct cutdown, and the interface of the cannula was functional end-to-end [5, 25]. In a patent application published in 2021, Flake et al. thoroughly illustrates its deliberations behind the umbilical cord cannulation technique including proposal regarding insertion technique, dilatation and fixation [26]. It resulted in breakthrough research of cannulation insertion in premature lambs (~4 weeks) [5, 27]. On the contrary, Charest-Pekeski et al. conducted research on premature piglets and performed various cannulation techniques to examine the best option, which resulted in a technique that combined incision with Seldinger-like introduction [7]. This resulted in a reduction of vessel trauma and the possibility to use bigger sized cannula. In the publications of the ex-vivo uterine environment (EVE) research from the group in Perth, the cannulation method was not elaborated [8, 9, 28]. Yet, the umbilical cord anatomy between lambs and humans differs significantly, as lambs have two veins and two arteries, whereas the human cord has one vein and two arteries. Hence, when working with lambs, it could be considered to connecting only one umbilical vein and artery instead of both, so that the other vessels remain connected to the placenta for oxygenation, limiting the interruption of oxygen delivery in lambs [7].

Altogether, cord cannulation technique is not always in detail documented in AAPT research. Thus, both end-to-end cannulation and side cannulation require further research as to what is preferred by professionals.

### Cannula fixation

In this study no consensus was achieved on the specific fixation method (such as sutures, glue, stent, balloon) [I1-12, F1-2]. Many AAPT studies do not describe their cannula fixation methods [8, 10, 29], whereas a few refer to sutures in animal experiments [5, 7, 25], without elaboration. Non-suture methods for anastomosis such as microsurgery and poloxamers (type of glue) have been reported in other medical applications [30–32]. Poloxamers are thought to minimize vessel manipulation and thus reduce damage to the wall [32]. Less preference was given to glue, as it was thought to be too weak to hold the vessel pressure to maintain the physiologic blood flow [F1-2]. In addition, fetal toxicity is mentioned as a problem when using glue [I8]. However, it does work fast [32]. Investigating glue has only been done in experimental animal-related studies [32]. Currently in Dutch medical centers, glue is only used in skin tissue repair and not in vessel anastomosis [33].

The Coupler system was also mentioned as it is a rapid automatic suture tool for vascular anastomosis [I2]. It is currently used in breast reconstruction to create anastomoses in small diameter veins [34]. Thus far, interviewee I2 mentions its successful use in plastic surgery, with its improved firmness and steadiness of sutures. However the system has limitations, as it can only be used in selective groups of vessels with specific wall thickness [21]. It is also questionable whether it can connect umbilical cord vessels and artificial cannulas as the material differs. Moreover, the thickness and//or diameter of umbilical cord vessels vary among patients, so compatibility with a large range in vessel sizes is a prerequisite [I2]. The use of the Coupler system has not been mentioned previously in AAPT-related animal studies and should be tested ex-vivo first.

### Anticoagulation

Interviewees expressed their concern regarding thrombosis and clotting risks [I3, I5, I6, I7, I12]. Whereas local anticoagulation is preferred, systemic anticoagulation is also thought to be necessary [F1]. Almost all experiments in lambs used continuous intravenous low-dose heparin due to the risk of thrombogenicity [5, 6, 8]. The doses were regulated based on an activated clotting time [5–8]. However, I4 was concerned about using activated clotting time due to the lack of accuracy and relatively large range of this factor. In this research, I3, I4, I7, I8, I11 are in favor of using coated cannulas, stents, or extracorporeal systems. This may prevent or at least reduce systemic anticoagulation, since coating may reduce clotting on foreign material [35]. As the interviews correctly mentioned; brain maturation of premature lambs occurs earlier than human brain maturation. The risks of intracranial hemorrhage cannot directly be translated to human extreme premature neonates [F1] [5]. In previous animal research, if local anticoagulation was used, tube or cannula-coating (in-and outflow plus oxygenators) was performed targeting different blood components, such an example is heparin [25, 36]. Literature illustrates another anticoagulation option, namely priming of the system with maternal blood [10, 25, 29]. This however increases blood volume, which, if it excesses placental reserve, could cause fetal physiological decompensation [36]. Both interviewees and literature prefer local to systemic anticoagulation; however, it is not clear whether local anticoagulation options have systemic effects [6].

### Vasospasm

Several interviewees expressed concerns regarding the effect of umbilical vasospasms on the success of cannulation [I1, I5, I10, I11, I12]. De Bie et al. also discussed in their review that vasospasm is one of the main vascular access challenges related to AAPT-based research and application [6]. If umbilical vessels are occluded, circulatory arrest and demises could occur [6]. The article suggested various ways to handle vasospasm via papaverine administration, moisturizing the vessels, by applying warmth, and the use of short umbilical cannulas [5, 6, 10].

Regarding papaverine, I5 is hesitant to its usage due to lack of experience as it is currently not used in obstetrical or neonatal Dutch clinics. Researchers working on peripheral arterial lines stated that papaverine could be effective to reduce vasospasm, but it should be used with caution in premature infants because of increased intracranial hemorrhages risks [37]. In all lamb experiments papaverine was given intravenous or topical [7, 25, 36]. No other medicaments were used to oppose vasospasm, such as lidocaine or nitroglycerine. Evidence of lidocaine’s effect on vasospasm is contradictory and may therefore not be used in lamb experiments [37]. In addition, nitroglycerine was discussed as vasodilator, but this has not been mentioned in lamb experiments [38, 39] and little experience was present within the experts [F1].

### Methodological considerations

In this qualitative study interviewees were sampled via snowball sampling. This is prone to selection bias since selection takes place only via the network of already involved participants. The participants were contacted via the project leader of the research group or through related colleagues. In total twelve experts with different clinical backgrounds participated. Not all were affiliated with AAPT-based research. All experts were based in the Netherlands, however many of them have working experience abroad and are part of international research groups; but they may not be completely familiar with the techniques used aboard.

The coding process was time consuming and therefore each interview and focus group was only coded by one researcher. The coding tree was debriefed by a peer with experience in qualitative research. After weekly discussions, the categories based on the coding process were formed.

Two qualitative data collection methods were integrated: interviews and focus groups. This research strived for completeness of data via interviews and for obtaining a deeper understanding of specific topics through discussion of preliminary results in the focus groups and to concluded further aspects that should be investigated. In this research two focus groups were conducted with three and four interviewees. The optimal size for a focus group is between five and twelve participants as too small groups limits the total range of experience [40, 41]. After the second focus group thematic saturation occurred, meaning that all categories had been formed and no new ones emerged. Therefore, no additional focus groups were scheduled.

## Conclusion

This study indicates that, for the required umbilical cord cannulation technique, less wall damage, better vessel stabilization, maintaining blood flow, tight fixation connection and local anticoagulation effects should be preferred. The next step would be to decide on cannulation technique as literature and experts demonstrate different opinions and experiences. Pilot experiments on the four topics (cannulation technique, fixation method, anticoagulation and vasospasm) should include human umbilical cord tissue.

## Supporting information

S1 File Coding tree focus groups. (XLSX)

S2 File Coding tree interviewees. (XLSX)

S3 File Quotes interviewees. (DOCX)

S1 Appendix (DOCX)

## Abbreviations

AAPT: artificial amnion and placenta technology
F1: focus group 1
F2: focus group 2
I1 - I12: interviewee 1 to interviewee 12
PLS: perinatal life support system. A liquid-based environment to mimic the natural womb to sustain fetal physiological development ex utero after extreme preterm birth
