# Supplementary material for: Clinical aspects of umbilical cord cannulation during transfer from the uterus to a liquid-based perinatal life support system for extremely premature infants a qualitative generic study
Source: PLoS One. 2023 Dec 21;18(12):e0290659. doi: 10.1371/journal.pone.0290659 (PMC10734990; doi:10.1371/journal.pone.0290659)
Supplement: S3 File — (DOCX) [file pone.0290659.s003.docx]

**S3 File. Box 1: quotes interviewees**

Quote 1: *“I think that: the advantage of that is that you keep the umbilical cord physiology, the usual reflexes and responses of the umbilical artery and vein constriction and the regulation of blood flow continuous that way” [I12].*

Quote 2: *“Gluing is possible with histoacryl glue. Sutures and clamps may also be possible. Even using a clamp is a challenge; it should be tight enough to hold its place. Or sutures can be wrapped around. It should not be so tight that it gives ischemia, because then it will die, and it will be loose in no time” [I11].*

Quote 3: *“You have to be aware that it is relatively tight of course, because that Wharton's jelly from the umbilical cord is loose, so if you pull hard enough, the sutures let go” [I5].*

Quote 4: *“You have to have clotting in order. Otherwise, you cannot use an artificial womb, because then you will have the same problems as we have with ECMO. You probably have a high chance of brain hemorrhages and co-morbidity and then the profit is lost” [7].*

Quote 5: *“Neonatologists get very restless with words like heparin. We think that’s deadly” [I5].*

Quote 6: *“You would actually prefer to have some sort of heparin coated cannula. So, you don't get clots on the walls” [I8].*

Quote 7: *“I think it's very difficult, because when such an artery is stimulated you get a spasm very quickly and then you just can't get in. Then you finished and the only thing is to mechanically dilate the vessel, and then insert your cannula” [I11].*
