# Supplementary material for: Clinical aspects of umbilical cord cannulation during transfer from the uterus to a liquid-based perinatal life support system for extremely premature infants a qualitative generic study
Source: PLoS One. 2023 Dec 21;18(12):e0290659. doi: 10.1371/journal.pone.0290659 (PMC10734990; doi:10.1371/journal.pone.0290659)
Supplement: S1 Appendix — (DOCX) [file pone.0290659.s004.docx]

Manuscript – Appendix 1 coding tree

**Appendix A** - Coding tree interviews

| **Main category** | **Groups** | **Code** | Groundedness | |  |
| --- | --- | --- | --- | --- | --- |
| Cannulation general | Cannulation general | ● Cannulate | 9 |  |  |
|  |  |  | ○ consider cannulation in different stages: first in the umbilical cord, then in another vessel | |  |
|  |  |  | ○ fetal surgeons do not puncture loose umbilical loop in utero | |  |
|  |  |  | ○ hard to say which way to puncture and cannulation umbilical cord vessel | |  |
|  |  |  | ○ in umbilical cord cannulation produce one artery and one vein is cannulated | |  |
|  |  |  | ○ individual cannulation, per vessel, is preferred | |  |
|  |  |  | ○ it is almost impossible to cannulate vessels one-by-one | |  |
|  |  |  | ○ preferably cannulation under water | |  |
|  |  |  | ○ puncturing before neonate is born (such as puncturing in fetal surgery) is a complicated situation | | |
|  |  |  | ○ think that EXIT procedure must be done: the umbilical cord is brought out of the womb without disconnecting the fetus | | |
|  |  | ● Cannulating too fast | 5 |  |  |
|  |  |  | ○ false route is created, when pushing cannula in umbilical cord during umbilical cord procedure | |  |
|  |  |  | ○ gently insert in artery, do not rush down | |  |
|  |  |  | ○ insert catheter gently otherwise forced route | |  |
|  |  |  | ○ too fast cannulation, then force-route, next to the lumen | |  |
|  |  |  | ○ when too fast it will swell | |  |
|  |  | ● Challenges in cannulation | 20 |  |  |
|  |  |  | ○ after cannulating vein, artery should follow directly, otherwise fetus/neonate is danger | |  |
|  |  |  | ○ always have an exit when there is an entry | |  |
|  |  |  | ○ artery should be punctured at the same time as vein | |  |
|  |  |  | ○ different challenges when puncturing under water (in bath): sight, fixation | |  |
|  |  |  | ○ difficult to cannulate underwater | |  |
|  |  |  | ○ eventually 70-80% of cases go into one of the two arteries, but not easy | |  |
|  |  |  | ○ experience shows that puncturing the umbilical cord is not that easy | |  |
|  |  |  | ○ if one artery fails, the other can be used | |  |
|  |  |  | ○ in lambs experiment cannulation was fixed within 2 minutes | |  |
|  |  |  | ○ in PLS keep fetus/neonate in liquid environment | |  |
|  |  |  | ○ increased risk when a loose vein is punctured | |  |
|  |  |  | ○ inserting a cannula before birth is very challenging, and will not be beneficial | |  |
|  |  |  | ○ ischemia is not well seen in PLS population as the fetus remains under water | |  |
|  |  |  | ○ it is more difficult to insert a cannula in unstable neonates/children | |  |
|  |  |  | ○ it would be ideal to cannulate all 3 umbilical vessels at the same time | |  |
|  |  |  | ○ more afraid of something happening in the neonate that can be observed | |  |
|  |  |  | ○ notorious complication of umbilical cord cannulation is thrombosis and portal hypertension | |  |
|  |  |  | ○ puncturing the right (small) vessel is not always easy | |  |
|  |  |  | ○ umbilical cord cannulation procedure does not always work in neonates | |  |
|  |  |  | ○ upon insertion of cannula/line artery pulsates more | |  |
|  |  | ● Characteristics of arteries | 10 |  |  |
|  |  |  | ○ arteries are smallest and roundest | |  |
|  |  |  | ○ arteries are thinner and two thin white lines are visible | |  |
|  |  |  | ○ arteries are very vulnerable | |  |
|  |  |  | ○ arteries can vasoconstrict | |  |
|  |  |  | ○ arteries have thicker wall, more constricted, rounder | |  |
|  |  |  | ○ arteries often lie in circles around the veins | |  |
|  |  |  | ○ arteries revolve around umbilical cord | |  |
|  |  |  | ○ diameter of an umbilical cord artery is a few millimeters | |  |
|  |  |  | ○ more to base: vein more towards cranial side and the two arteries more caudal | |  |
|  |  |  | ○ neonatologist often starts with artery due to complexity | |  |
|  |  | ● Characteristics of veins | 11 |  |  |
|  |  |  | ○ depending on where the umbilical cord is cut, the vein lies caudal or cranial | |  |
|  |  |  | ○ inserting a venous catheter is often not the problem, because there is enough space, but it bleeds the most | | |
|  |  |  | ○ the umbilical vein is vulnerable; thin-wall vessel | |  |
|  |  |  | ○ umbilical wall vessel is more fragile than vessel in the body | |  |
|  |  |  | ○ vein is larger and darker blue compared artery | |  |
|  |  |  | ○ vein is weaker | |  |
|  |  |  | ○ vein is wide blue line | |  |
|  |  |  | ○ vein is wider than artery, around 2-3mm | |  |
|  |  |  | ○ vein is wider, more often on the caudal side | |  |
|  |  |  | ○ vena is thin and larger than artery | |  |
|  |  |  | ○ vena is vulnerable | |  |
|  |  | ● Depth insertion cannula | 22 |  |  |
|  |  |  | ○ can spasm occur when catheter is not inserted deep enough? not sure | |  |
|  |  |  | ○ cannulation in umbilical cord vessels in neonates is difficult (up to iliaca) due to curve of the vessel | | |
|  |  |  | ○ catheter should preferably be placed just below the diaphragm, otherwise there is a risk of renal infarction | | |
|  |  |  | ○ deepen cannula to ensure proper blood sampling for monitoring | |  |
|  |  |  | ○ do not belief in catheter should only be placed in umbilical cord | |  |
|  |  |  | ○ if cannula is placed before the liver, medication go directly into the liver and not the rest of the body | | |
|  |  |  | ○ in 30% of cases, venous cannulation goes into portal vein | |  |
|  |  |  | ○ in animal experiments the cannula was not inserted up to the abdominal wall | |  |
|  |  |  | ○ in current neonatal cannulation setting: the umbilical cord cannula is placed as close to the heart as possible | | |
|  |  |  | ○ In fetal surgery, the umbilical cord is punctured at the root of the umbilical cord near the placenta because of support from surrounding tissue | | |
|  |  |  | ○ in neonates; if central line did not work, a peripheral line was placed | |  |
|  |  |  | ○ in theory, do not place the cannula all the way to the abdominal wall opening | |  |
|  |  |  | ○ inserting a small piece of the cannula does not lead to a longer insertion | |  |
|  |  |  | ○ one artery is punctured, as there is no room for advancing 2 lines | |  |
|  |  |  | ○ preserve umbilical cord physiology by inserting only a little bit | |  |
|  |  |  | ○ to draw blood the cannula should be placed deep | |  |
|  |  |  | ○ umbilical stump should not be to long when cannulating | |  |
|  |  |  | ○ vessel cannulation in the first 5 cm is okay, but after that it becomes increasingly difficult | |  |
|  |  |  | ○ when cannula is only inserted a little bit, be careful with giving medication because you can induce damage | | |
|  |  |  | ○ when inserting only a little piece of cannula, there is a high risk of failure and bleeding of the neonate/child | | |
|  |  |  | ○ when vein cannulation is placed just before the liver, all the given medication goes first to the liver instead of the rest of the body | | |
|  |  |  | ○ with only little cannulation, the umbilical cord will remain its function as it is still in fetal phase (not neonatal phase) | | |
|  |  | ● Dilate vessel | 11 |  |  |
|  |  |  | ○ a break seam can be used through with the catheter can pass | |  |
|  |  |  | ○ dilate vessel with tweezers | |  |
|  |  |  | ○ dilating vessels causes wall damage | |  |
|  |  |  | ○ gently dilate vessel opening with alternating leg tweezers, so that the vessel is stretched a bit | |  |
|  |  |  | ○ in such small children, vessels are stretchy and do not tear fast | |  |
|  |  |  | ○ in umbilical cannulation procedure an introducer (often a wide needle) can be used, one that is larger than the vessel | | |
|  |  |  | ○ open vessel wider with tweezers | |  |
|  |  |  | ○ slide dilator with sheath over guidewire; and then remove dilator with sheath | |  |
|  |  |  | ○ stretching the umbilical cord would be possible, but you risk damage and vasoconstriction | |  |
|  |  |  | ○ when dilating first 5mm is used, another 1-1.5cm is undamaged vessel remains | |  |
|  |  |  | ○ with tweezers open the vessel a bit | |  |
|  |  | ● Echo/doppler usage | 8 |  |  |
|  |  |  | ○ at 25 weeks an ultrasound doppler with small probe | |  |
|  |  |  | ○ doppler can be used to distinguish pressure | |  |
|  |  |  | ○ doppler: use warm gel or maybe nothing because it is wet | |  |
|  |  |  | ○ fetal surgeons monitor fetus via ultrasound when transfusion occurs | |  |
|  |  |  | ○ fetal surgeons often use ultrasound, and everything is made as small as possible | |  |
|  |  |  | ○ in fetal surgeon, the crux of the examination is mapping the position of the fetus, where is the umbilical cord, etc., all with an ultrasound | | |
|  |  |  | ○ mini-doppler can give us information on whether the blood vessel is still open or that it is closed | |  |
|  |  |  | ○ with echo doppler you are directly under the surface; so high frequency with regular small vessel doppler | | |
|  |  | ● Hold vessel | 8 |  |  |
|  |  |  | ○ when grabbing umbilical cord and vessel is not grabbed than an option would be to grab the vessel in a tweezer | | |
|  |  |  | ○ bond sutures through Wharton's jelly away to the side of the vessel, have someone fixate it, and then potentially attach a device for optimal lumen vision | | |
|  |  |  | ○ by grabbing umbilical cord, try not to squeeze or grab vessel | |  |
|  |  |  | ○ fixating vessel by grabbing Wharton jelly with a tweezer | |  |
|  |  |  | ○ fixating vessel on the side in the Wharton jelly, turn the vessel a bit upwards so that the opening of the vessel is good visible | | |
|  |  |  | ○ fixation vessel by grabbing artery wall with tweezer, however too much traction causes release | |  |
|  |  |  | ○ holding umbilical cord only with hands | |  |
|  |  |  | ○ preferred optimal presentation of vessels to increase success rate | |  |
|  |  |  | ○ vessels in premature infants are so small that a tweezer must hold the vessel | |  |
|  |  | ● Peripheral cannulation | 9 |  |  |
|  |  |  | ○ EXIT-to-EMCO cannula is inserted in the neck, the neonate goes directly from the placenta to ECMO | | |
|  |  |  | ○ hematoma formation when puncturing inguinal | |  |
|  |  |  | ○ if the foot is out, then it may be possible to puncture vena saphenous magma; previously used for long lines | | |
|  |  |  | ○ in EXIT-to-EMCO, coronary artery and jugular vein are used | |  |
|  |  |  | ○ is it possible to put a foot outside, for artery and vein access? | |  |
|  |  |  | ○ neck vessels are used, because vessels in the extremities have increased risk of ischemia, resulting in leg necrosis | | |
|  |  |  | ○ neonatologist: other lines via Seldinger | |  |
|  |  |  | ○ should the umbilical cord be used? Most obvious though | |  |
|  |  |  | ○ when inserting lines through the elbow, a breaking needle (= introducer) is used, before the cannula | | |
|  |  | ● Usage of disinfectants | 7 |  |  |
|  |  |  | ○ disinfectant can also have a vasoconstrictive effect | |  |
|  |  |  | ○ disinfectant could cause dehydration of tissue | |  |
|  |  |  | ○ in PLS: also sterilize with vaginal birth? | |  |
|  |  |  | ○ instead of disinfectant, rinsing with warm/sterile water may be better | |  |
|  |  |  | ○ umbilical cord stump is disinfected for first cannulation | |  |
|  |  |  | ○ not need to sterilize with a PLS caesarean, since everything is probably already sterile | |  |
|  |  |  | ○ special sterile umbilical cannulation set is prepared in advance | |  |
|  |  | ● Vessel damage | 4 |  |  |
|  |  |  | ○ cannula has a fixed diameter, resulting in more endothelial damage when inserting | |  |
|  |  |  | ○ guide wire can be friendly to intima | |  |
|  |  |  | ○ very small caliber peripheral central venous cannula with (metal) flexible tip is probably not harmful | | |
|  |  |  | ○ when the cannula is inserted, wall damage will only occur in the first few millimeters; the tip of the cannula ends in a relatively undamaged part of the vessel | | |
|  |  | ● Vessel recognition | 18 |  |  |
|  |  |  | ○ arteries and vein are distinguished by the experienced eye | |  |
|  |  |  | ○ arteries and vein not always easy to distinguish | |  |
|  |  |  | ○ at some point everyone punctured and cannulated the wrong vessel | |  |
|  |  |  | ○ distinguish vein/artery via pressure measurement | |  |
|  |  |  | ○ echo doppler can be used to differentiate between venous and arterial | |  |
|  |  |  | ○ good orientation with the ultrasound before transition is important | |  |
|  |  |  | ○ idea: to develop a device that recognizes an artery or vein | |  |
|  |  |  | ○ ideally, vessels should be punctured via some kind of echo conductor | |  |
|  |  |  | ○ inspection vessel; most of the time things go well, but things can also go wrong, even in the hands of someone experienced | | |
|  |  |  | ○ it is beneficial to make an ultrasound before intervention: to view the umbilical cord and to properly estimate size / shape / jelly thickness | | |
|  |  |  | ○ pressure measurement for vessel recognition/position checking is not used, only during checks | |  |
|  |  |  | ○ recognize and differentiate vessels depends on where the umbilical cord is cut | |  |
|  |  |  | ○ recognizing vessels depends on combination of cutting off location and seeing what they look like | |  |
|  |  |  | ○ remains a challenge to be able to properly differentiate vessels | |  |
|  |  |  | ○ thickness of veins and arteries and number of rotations can be seen on ultrasound | |  |
|  |  |  | ○ to the trained eye, arterial blood looks different from venous blood | |  |
|  |  |  | ○ vessel recognition by eye | |  |
|  |  |  | ○ visual inspection distinguishes artery from vein | |  |
| Cannulation technique | Cannulation via side-entry technique | ● Benefit side-entry (English technique) | 4 |  |  |
|  |  |  | ○ cannulation is faster with the English method | |  |
|  |  |  | ○ in Utrecht assistants experience side approach as much easier and nicer | |  |
|  |  |  | ○ side-entry approach is faster; easier and success rate is higher than transversal dissection | |  |
|  |  |  | ○ via side-entry cannulation; no vasospasm was seen | |  |
|  |  | ● Benefit side-entry (Seldinger technique) | 11 |  |  |
|  |  |  | ○ advantage of whole umbilical cord cannulation is that physiology, reflexes, responses, and regulation of blood flow are maintained | | |
|  |  |  | ○ advantage: if the side-entry approach does not work, you can always cut through later | |  |
|  |  |  | ○ it is logical to make a mechanical connection; insert tube into blood vessel | |  |
|  |  |  | ○ most elegant is to puncture lumen with ultrasound guidance and insert the cannula (Seldinger type) | | |
|  |  |  | ○ puncturing is more common; it gives you more time | |  |
|  |  |  | ○ Seldinger technique is cannulation option, to quickly introduce catheters | |  |
|  |  |  | ○ Seldinger: perhaps faster, vessel-by-vessel | |  |
|  |  |  | ○ side incision does not withdraw easy | |  |
|  |  |  | ○ side-entry approach only takes like 5 minutes; really fast method | |  |
|  |  |  | ○ side-entry cannulation is not definitive, so if it does not work you can try another option | |  |
|  |  |  | ○ the advantage of sideways cannulation can be if only one vessel is cannulated first, meaning more time for the rest later | | |
|  |  | ● Disadvantages of side-entry cannulation (Seldinger technique) | 12 |  |  |
|  |  |  | ○ 1mm too thin to puncture via Seldinger | |  |
|  |  |  | ○ advance guidewire in Seldinger technique may damage endothelium, but does not always have to be the case | | |
|  |  |  | ○ advancing the guidewire may cause endothelial damage | |  |
|  |  |  | ○ difficult to cannulate with side-incision | |  |
|  |  |  | ○ disadvantage of Seldinger cannulation is that it takes a lot of time | |  |
|  |  |  | ○ fiddle with tube from artificial placenta as it is all very small | |  |
|  |  |  | ○ for experience: when puncturing the pH sideways, the blood vessel is often misjudged | |  |
|  |  |  | ○ if pH puncture is already difficult, how can side-entry punction succeed | |  |
|  |  |  | ○ neonatologists lack experience and skills with Seldinger technique in very small vessels | |  |
|  |  |  | ○ Seldinger technique is also used in fetal surgery, but it takes more time | |  |
|  |  |  | ○ Seldinger technique is difficult with small vessels | |  |
|  |  | ● Current usage of Seldinger technique | 2 |  |  |
|  |  |  | ○ in neonate Seldinger is used for femoral puncture | |  |
|  |  |  | ○ in Seldinger technique vessel is punctured without strong traction | |  |
|  |  | ● General: Seldinger technique | 4 |  |  |
|  |  |  | ○ neonatologists prefer umbilical cord cannulation when the cord is still attached to the placenta | |  |
|  |  |  | ○ Seldinger technique would be nice, only if there is little damage to endothelium | |  |
|  |  |  | ○ side port is also option for dilator and guide, as end-port can be placed | |  |
|  |  |  | ○ the order of Seldinger technique is important, as we do not want anyone to bleed tremendously | |  |
|  |  | ● Side-entry cannulation actions | 5 |  |  |
|  |  |  | ○ blade should be held parallel to the vessel | |  |
|  |  |  | ○ fetal surgeon: do not cut, but puncture umbilical cord | |  |
|  |  |  | ○ possible to create side-entry for the guide wire | |  |
|  |  |  | ○ start cannulation when vessel is tightened around the finger | |  |
|  |  |  | ○ traction on umbilical cord by placing it over one's finger. Then also differentiate between artery and vein | | |
|  | Cannulation via transversal dissection | ● Disadvantages of transversal dissection | 9 |  |  |
|  |  |  | ○ complete transversal dissection causes more vasospasm | |  |
|  |  |  | ○ much more labor-intensive and takes longer | |  |
|  |  |  | ○ hard to see vessel opening after transversal dissection | |  |
|  |  |  | ○ the disadvantage with a complete cross-section is that the intima withdraws, making the lumen difficult to find | | |
|  |  |  | ○ to do a complete transection, the blood vessels must first stop bleeding | |  |
|  |  |  | ○ transversal dissection has a great risk, namely blood flow interruption | |  |
|  |  |  | ○ transversal dissection is resolute method | |  |
|  |  |  | ○ when cut off, the umbilical artery can curl up a bit | |  |
|  |  |  | ○ when transversal dissection (complete cut off umbilical cord) a time limit should be established | |  |
|  |  | ● Benefits of transversal dissection | 6 |  |  |
|  |  |  | ○ blood vessels are recognizable on coronary/cross-section plane | |  |
|  |  |  | ○ easy to recognize a cross-section of vessels | |  |
|  |  |  | ○ in transversal dissection more visibility | |  |
|  |  |  | ○ sometimes it is necessary to re-cut transversal dissection due to unclear presentation of lumen | |  |
|  |  |  | ○ the preferred option is to do it right at ones | |  |
|  |  |  | ○ with complete transection; best view and best chance of success | |  |
| Cannula | Cannula | ● Current cannula size | 17 |  |  |
|  |  |  | ○ 1 French is about 3mm | |  |
|  |  |  | ○ 3.5French for both artery and vein | |  |
|  |  |  | ○ 6 French can be added retrograde | |  |
|  |  |  | ○ 8-10 French arterial and venous used for 2-2.5kg | |  |
|  |  |  | ○ 22 Gaunch is not equivalent with diameter of normal vessel | |  |
|  |  |  | ○ 22 Gaunch is used in fetal surgery, but it is to small for continuous blood flow | |  |
|  |  |  | ○ cannula in artery is 3-4 French | |  |
|  |  |  | ○ fetal surgeon: puncture needle is 22 or 20 Gaunch | |  |
|  |  |  | ○ fetal surgeons use needles, not catheters | |  |
|  |  |  | ○ for vein cannula 4 French is the smallest, but then a good flow is already a hassle | |  |
|  |  |  | ○ in little ones under 28 weeks 3.5 French is used | |  |
|  |  |  | ○ in neonate vein line is 5 French | |  |
|  |  |  | ○ largest cannula: 5 French is 1.67mm | |  |
|  |  |  | ○ smallest cannula is 2.5 French for arterial line and 3.5-4-5-French umbilical vein line | |  |
|  |  |  | ○ smallest: 2.5 French is 0.8mm | |  |
|  |  |  | ○ smallest: 8 French arterial and 10 French venous | |  |
|  |  |  | ○ vascular surgeon does not use small cannulas | |  |
|  |  | ● Example catheter/cannula | 7 |  |  |
|  |  |  | ○ advantage HERO: the cannula of the artificial womb can already be made | |  |
|  |  |  | ○ advantage of HERO is that it consists of 2 parts, which means that it has the most flexible | |  |
|  |  |  | ○ in HERO cannulation extra blood vessel prepared to cannulate | |  |
|  |  |  | ○ HERO cannula is used for dialysis when cannulating large vessels | |  |
|  |  |  | ○ HERO is expensive | |  |
|  |  |  | ○ HERO is used in all major dialysis hospital in the Netherlands | |  |
|  |  |  | ○ HERO provides high flow which is important in extra-corporeal circulation | |  |
|  |  |  | ○ in hemodialysis there is experience with heparin-free hemodialysis with citrate | |  |
|  |  | ● Length cannula | 6 |  |  |
|  |  |  | ○ 2cm cannula could come loose | |  |
|  |  |  | ○ 2cm in vessel seems logical | |  |
|  |  |  | ○ cannula is not measured before use; the size is estimated | |  |
|  |  |  | ○ length of 2cm is nice, but shorter might be better | |  |
|  |  |  | ○ risk of loosening with shorter cannula length | |  |
|  |  |  | ○ vascular surgeons use range of stents for the coronary vessels. the smallest is 5mm | |  |
|  |  | ● Material cannula | 9 |  |  |
|  |  |  | ○ catheter consists of plastic-like material | |  |
|  |  |  | ○ do not know why a double lumen should be used | |  |
|  |  |  | ○ ideal circumstances would be a short catheter, little foreign material in the body, anti-coagulant coating, without the release of heparin | | |
|  |  |  | ○ in vena cannulation, double lumen cannula was used | |  |
|  |  |  | ○ option is smoothening the tip of the cannula, but you still may touch vessel wall | |  |
|  |  |  | ○ other tubes can stay in longer than 2.5 days, but they are not placed in artery or vein | |  |
|  |  |  | ○ part of PFTE-tube can work as cannula, but very thin material | |  |
|  |  |  | ○ silicone is most used, but polyurethane material is also used for long lines | |  |
|  |  |  | ○ small market for producing cannulas and thus expensive | |  |
|  |  |  | ○ vascular surgery materials are not suitable for maintaining long-term blood circulation | |  |
|  |  | ● Requirements cannula | 13 |  |  |
|  |  |  | ○ a long umbilical cord is required for side approach cannulation; this is a question for the obstetrician | | |
|  |  |  | ○ artificial umbilical cord cannula should completely be prepared | |  |
|  |  |  | ○ cannula must be flexible due to umbilical cord rotations | |  |
|  |  |  | ○ cannula should be in place a long period of time, not just one hour | |  |
|  |  |  | ○ entire cardiac output needs to go through cannula; thus, it must be a good caliber cannula | |  |
|  |  |  | ○ important: what is the resistance of blood? what size should the diameter be? | |  |
|  |  |  | ○ it is important to generate enough flow | |  |
|  |  |  | ○ it is important to know how much blood must go through the cannula/line | |  |
|  |  |  | ○ it is important to prevent any nods in the vessel | |  |
|  |  |  | ○ must have adequate venous flow | |  |
|  |  |  | ○ short cannula is preferred so less foreign material is introduced | |  |
|  |  |  | ○ use of tapered tip catheter for good seal | |  |
|  |  |  | ○ you can calculate which flow you should run, and then see which cannula you need to generate this flow | | |
| Physiology | Physiology | ● Amniotic fluid | 2 |  |  |
|  |  |  | ○ in clear amniotic fluid, gasping is not a problem, when still attached to the original umbilical cord | |  |
|  |  |  | ○ when fetus/neonate is taken out of amnionic fluid, it is done | |  |
|  |  | ● Apgar score | 3 |  |  |
|  |  |  | ○ Apgar score is subjective | |  |
|  |  |  | ○ gyneacologists give higher Apgar score compared to neonatologists | |  |
|  |  |  | ○ pediatricians give lower Apgar scores in the beginning and higher afterwards | |  |
|  |  | ● Blood flow | 13 |  |  |
|  |  |  | ○ adults can maintain cerebral flow well, preterm infants cannot | |  |
|  |  |  | ○ flow is limited by vessel diameter | |  |
|  |  |  | ○ flowrate in placenta in unclear | |  |
|  |  |  | ○ it is unclear whether vessel traction is sufficient to prevent blood backflow | |  |
|  |  |  | ○ knowledge about in- and outflow has been gathered with exchange transfusion | |  |
|  |  |  | ○ less flow needed in fetal physiology than in neonatal physiology | |  |
|  |  |  | ○ maybe flow not enough for neonate (~1kg) with cannula of 2 French | |  |
|  |  |  | ○ one of the limitations is venous flow | |  |
|  |  |  | ○ placenta lets almost everything through without pumping, so expect equal arterial and venous pressures | | |
|  |  |  | ○ premature infants lack autoregulation | |  |
|  |  |  | ○ push-and-pull system puts blood in and takes blood out | |  |
|  |  |  | ○ running flow, for example: 180 ml/kilogram for a neonate of 2-2.5 kg | |  |
|  |  |  | ○ venous flow depends on age | |  |
|  |  |  | ○ with complete cannulation takeover, venous flow is restrictive and arterial less restrictive | |  |
|  |  | ● Blood pressure | 5 |  |  |
|  |  |  | ○ dobutamine is for blood pressure, that it remains normal | |  |
|  |  |  | ○ dobutamine is given for good blood pressure | |  |
|  |  |  | ○ premature infants are extremely sensitive to blood pressure changes | |  |
|  |  |  | ○ rapid disturbance of blood pressure leads to faster cerebral hemorrhages | |  |
|  |  |  | ○ small change in blood pressure causes increased flow in the brain | |  |
|  |  | ● Circulation | 7 |  |  |
|  |  |  | ○ artery will also bleed heavily when cannulated with intact placental umbilical circulation | |  |
|  |  |  | ○ catheter can cause poor circulation in the leg | |  |
|  |  |  | ○ discussion stays, will it remain a fetus or will it become a neonate | |  |
|  |  |  | ○ in PLS the pumping heart maintains circulation | |  |
|  |  |  | ○ it is a shame to let the fetus/neonate bleed out, only to add donor blood | |  |
|  |  |  | ○ keep circulation and oxygenation interruptions to a minimum | |  |
|  |  |  | ○ people are very hesitant to give fluid to premature neonates due to increased risk of cerebral hemorrhages | | |
|  |  | ● Condition neonate/perinate | 14 |  |  |
|  |  |  | ○ 500 grams has 40 mL of blood | |  |
|  |  |  | ○ after 5 minutes there is a problem if nothing happens in terms of cannulation | |  |
|  |  |  | ○ already restless after 1 min in bradycardia | |  |
|  |  |  | ○ it is preferred that with bradycardia during birth the heartbeats need to restore | |  |
|  |  |  | ○ cannot say how long the neonate can remain attached to the umbilical cord outside the uterus | |  |
|  |  |  | ○ CTG is useless at the very last moment; point of no-return | |  |
|  |  |  | ○ gasping is the result of drowning, and that happens very quickly | |  |
|  |  |  | ○ CTG between 130-160 bpm is preferred, little lower/higher is acceptable | |  |
|  |  |  | ○ if foot is out for cannulation, do not know what will happen to the foot | |  |
|  |  |  | ○ if not punctured, neonate will gasp | |  |
|  |  |  | ○ if the child at the start is in good condition, it can handle a little more stress | |  |
|  |  |  | ○ increasing CO2 and breathing stimulus | |  |
|  |  |  | ○ neonate cannot tolerate bradycardia for a long time | |  |
|  |  |  | ○ neonates are clearly oxygenated and pink, in PLS system this may not be the case and more difficult to see | | |
|  |  |  | ○ respiratory stimuli: 1) cooling (thus cold), 2) clamping umbilical cord as saturation that goes down | | |
|  |  |  | ○ when still in the uterus, fetuses cab withstand hypoxia for longer period, outside the uterus is a thermo-hostile environment, which causes a lot of energy loss | | |
|  |  | ● Emergency procedure | 1 |  |  |
|  |  |  | ○ if cannulation fails, the neonate should be helped in the conventional method | |  |
|  |  | ● pH | 6 |  |  |
|  |  |  | ○ many pH measurements were missed, especially in stress situations | |  |
|  |  |  | ○ pH of 7.35 indicates good condition of neonate | |  |
|  |  |  | ○ pH value is more objective | |  |
|  |  |  | ○ puncture pH, but no cannula is left inside the vessel | |  |
|  |  |  | ○ puncture pH side-ways is not that difficult | |  |
|  |  |  | ○ umbilical cord pinching causes pH drop per minute | |  |
|  |  | ● Resistance | 1 |  |  |
|  |  |  | ○ a resistance-free circulation is preferred | |  |
|  |  | ● Saturation | 8 |  |  |
|  |  |  | ○ Dawson curves for saturation ranges | |  |
|  |  |  | ○ Dawson curves shows normal development of heart rate and saturation in the first 5 min | |  |
|  |  |  | ○ fetal saturation is 75%, so they have blue color | |  |
|  |  |  | ○ intra-uterine saturation of 70% | |  |
|  |  |  | ○ neonates can handle low saturation post-partum | |  |
|  |  |  | ○ saturation and cold important for respiratory stimulus, especially CO2 | |  |
|  |  |  | ○ saturation of 60% does not make me restless, bradycardia up to 60 (2-3min) does make me restless | | |
|  |  |  | ○ why is saturation of 70% intra-uterine fine, and extra-uterine (once born) a problem? | |  |
|  |  | ● Warmth | 10 |  |  |
|  |  |  | ○ cold dry air causes constriction | |  |
|  |  |  | ○ cold, clenching, or squeezing does not cause a lot of vasospasm, compared to complete dissection | | |
|  |  |  | ○ for the PLS system: no exposure to dry and cold, but to hot and humid air | |  |
|  |  |  | ○ heat lamps are focused on neonate, to keep neonate warm, not to make access easier | |  |
|  |  |  | ○ it is important to keep warm, warm liquids, constantly sprinkling with warm liquid; always keep tissue wet | | |
|  |  |  | ○ it is important to not let the fetus/neonate cool down, and thus keep warm | |  |
|  |  |  | ○ temperature in the room can increase with heat lamps | |  |
|  |  |  | ○ the stress related to delivery requires a lot of energy from the fetus | |  |
|  |  |  | ○ warm blankets are used | |  |
|  |  |  | ○ warm cloth for vasodilation was put on leg | |  |
|  |  |  | ○ warm environment, no compression of umbilical cord, humid | |  |
| Cannula fixation | Usage of balloons | ● Benefits of using balloon | 4 |  |  |
|  |  |  | ○ balloon is possible, gives a nice watertight seal, but reduces the diameter | |  |
|  |  |  | ○ in vessel preparation the vessel is stretched so that the balloon can sit in the crack that arose | |  |
|  |  |  | ○ inflate the balloon and cannulate vessel should be possible within 2 minutes | |  |
|  |  |  | ○ easiest would be cannulation with stent, and then inflating the balloon | |  |
|  |  | ● Disadvantages of balloons | 5 |  |  |
|  |  |  | ○ disadvantage of balloon cannula is that it needs space in the vessel and thus decreased vessel diameter | | |
|  |  |  | ○ less blood goes through a tube with a balloon than through a tube with a thickened bud | |  |
|  |  |  | ○ permanent inflating a balloon in an umbilical artery or vein does not seem good | |  |
|  |  |  | ○ with balloon pressure is in one spots, whereas with stent it is divide over a bigger surface | |  |
|  |  |  | ○ with balloon-expendable stent an extra action is required, while you want it as short as possible in terms of time | | |
|  |  | ● Usage of balloons | 3 |  |  |
|  |  |  | ○ inserting cannula with balloon into the vessel is a possibility | |  |
|  |  |  | ○ option is to use balloon stents | |  |
|  |  |  | ○ with balloon-expandable stents, inflation of the balloon forces the stent to open and fixate in vessel | | |
|  | Usage of glue | ● Benefits of using glue | 3 |  |  |
|  |  |  | ○ blood vessel can be closed with histoacryl and lipiodol over it | |  |
|  |  |  | ○ glue is cheap | |  |
|  |  |  | ○ the advantage of cyanoacrylate is that it is very cheap | |  |
|  |  | ● Disadvantages of glue | 19 |  |  |
|  |  |  | ○ arteries have too much tension that is will not be held together with glue | |  |
|  |  |  | ○ cyanoacrylate is normally used on the skin, then difficult to get off | |  |
|  |  |  | ○ disadvantage of glue, especially histoacryl, is that it hardens quickly | |  |
|  |  |  | ○ enormous pressure ensures that gluing will not work | |  |
|  |  |  | ○ glue can be a temporary fixation, not used as permanent | |  |
|  |  |  | ○ glue has to dry at some point | |  |
|  |  |  | ○ glue is not a solution for cannulation | |  |
|  |  |  | ○ glue is very fragile, so not useful | |  |
|  |  |  | ○ glue may be fetotoxic, it has been investigated, but results were very unclear and complicated | |  |
|  |  |  | ○ glue was not used | |  |
|  |  |  | ○ glue was not used and not thought of | |  |
|  |  |  | ○ glue will not get arteries together | |  |
|  |  |  | ○ gluing vessel walls with polymers can be tricky, as on one side artificial vessel and the other side human vessel | | |
|  |  |  | ○ in damp conditions/humidity, gluing does not work | |  |
|  |  |  | ○ little experience with glue | |  |
|  |  |  | ○ poloxamers can be used at certain temperatures | |  |
|  |  |  | ○ research on cyanoacrylate has been done for years | |  |
|  |  |  | ○ with a moist surface you need something else besides cyanoacrylate | |  |
|  |  |  | ○ with histoacryl there is a chance that the material is stuck and not removable | |  |
|  |  | ● Current usage of glue | 11 |  |  |
|  |  |  | ○ blood vessels are not glued, skin is | |  |
|  |  |  | ○ Delf university has glue that works in a wet environment, but it still may be fetotoxic | |  |
|  |  |  | ○ fetal surgeons do nothing with the whole in the amnionic sac | |  |
|  |  |  | ○ glue is only used to close vessel in emergency setting | |  |
|  |  |  | ○ if glue works in different specialties, why can it not be used? | |  |
|  |  |  | ○ radiologists use glue to make things really tight for specific things | |  |
|  |  |  | ○ tissue gel can be used at the insertion site of the cannula | |  |
|  |  |  | ○ tissue glue is sometimes used when there is more than average blood loss; after an introducer is followed by cannula | | |
|  |  |  | ○ tissue glue is used close openings | |  |
|  |  |  | ○ use of poloxamers on moist surface | |  |
|  |  |  | ○ UV light is also used to harden the super glue | |  |
|  | Usage of stents | ● Benefits of using stents | 11 |  |  |
|  |  |  | ○ a covered stent does not leak | |  |
|  |  |  | ○ advantage of the stent is that it is very small at the time of insertion, so that it is not damage endothelial | | |
|  |  |  | ○ disadvantage of a plastic shunt is that it closes after a while | |  |
|  |  |  | ○ stent can remain in place according to vascular surgeon | |  |
|  |  |  | ○ stents could remain open for several years | |  |
|  |  |  | ○ the advantage of a stent is that it expands and clamps in the vessel | |  |
|  |  |  | ○ there are stents that counteract intimal hypertrophy which is a complication of stents | |  |
|  |  |  | ○ vascular surgeon thinks a stent would be a good method | |  |
|  |  |  | ○ vessel wall damage seems to be less than expected when using a stent | |  |
|  |  |  | ○ with a self-expandable stent, the stent pops open by itself | |  |
|  |  |  | ○ with a stent that consists of 2 parts, the first part can be prepared in advanced | |  |
|  |  | ● Disadvantages of stents | 7 |  |  |
|  |  |  | ○ disadvantage stent is possible vessel wall damage | |  |
|  |  |  | ○ only stents are not enough, suture on the outside are required | |  |
|  |  |  | ○ side-effect of stent is its unclear effect on epithelium and damage because | |  |
|  |  |  | ○ stent can cause irritation and possible thrombosis | |  |
|  |  |  | ○ stents are expensive | |  |
|  |  |  | ○ stents are not used in plastic surgery | |  |
|  |  |  | ○ there is a risk of intimal hyperplasia when using stents | |  |
|  |  |  | ○ when neonates grow, the vessel also grows, and thus the caliber stents should also be adjusted | |  |
|  |  | ● Usage of stents | 7 |  |  |
|  |  |  | ○ CARIM in Maastricht: may have thoughts on how the stents stay best open | |  |
|  |  |  | ○ contact GORE company about stent options | |  |
|  |  |  | ○ covered stents are required to keep blood vessels open | |  |
|  |  |  | ○ coated stents have metal core that is covered with something to prevent blood leakage | | |
|  |  |  | ○ ideally the stent should not be exposed but always covered with either artificial umbilical cord or normal umbilical cord | | |
|  |  |  | ○ stent is coated with PTFE/Dacron | |  |
|  |  |  | ○ stent could end partly in umbilical cord, partly in cannula | |  |
|  |  |  | ○ the stent should not leak | |  |
|  | Usage of sutures | ● Benefits of using sutures | 5 |  |  |
|  |  |  | ○ possible to make clean cut | |  |
|  |  |  | ○ it makes sense to suture vessel to tube after cannula placement for long-term fixation | |  |
|  |  |  | ○ no suturing of the cannula in the blood vessel, but sutures or clips on the outside, which gives pressure and ensures a watertight seal | | |
|  |  |  | ○ sutures are best chance for good fixation without ischemia | |  |
|  |  |  | ○ temporary clamp can be placed around the cannula in the umbilical cord and then quickly place sutures around it | | |
|  |  |  | ○ worries that cannula will fall out, that is why additional sutures are important | |  |
|  |  | ● Disadvantages of sutures | 2 |  |  |
|  |  |  | ○ perhaps Flake describes duration of suturing | |  |
|  |  |  | ○ suturing blood vessel timewise goes more towards 12 minutes, and we don't have that time | |  |
|  |  | ● Current usage of sutures | 12 |  |  |
|  |  |  | ○ all lines in the IC are sutured | |  |
|  |  |  | ○ attach to umbilical stump offers more stability | |  |
|  |  |  | ○ cannula is sutured to umbilical cord | |  |
|  |  |  | ○ catheter is sutured in Wharton's jelly | |  |
|  |  |  | ○ fetal surgeon has no experience with suturing and its related time | |  |
|  |  |  | ○ in plastic surgery, arteries are sutured by hand | |  |
|  |  |  | ○ neonatologists make sutures around umbilical cord & stump, pouch seam | |  |
|  |  |  | ○ sutures are not so tight due to Wharton's jelly | |  |
|  |  |  | ○ sutures are placed through Wharton jelly | |  |
|  |  |  | ○ sutures were placed around cannula and connected to the stump | |  |
|  |  |  | ○ suturing through the skin is not an option these days because it is too painful | |  |
|  |  |  | ○ the puncture in umbilical cord is too small to suture | |  |
|  | Other fixation options | ● Additional fixation | 1 |  |  |
|  |  |  | ○ glue with histoacryl, suture is possible, clip is possible: must be tight enough | |  |
|  |  | ● Coupler system | 23 |  |  |
|  |  |  | ○ advantage of coupler system is that it is less time consuming and the differentiation between calibers is not a problem | | |
|  |  |  | ○ Coupler system is easy for the vein, the artery in breast reconstruction operations is the problem | | |
|  |  |  | ○ coupler system has previously been used in arteries, but it was not a success due to its wall | |  |
|  |  |  | ○ Coupler system work with click and turn system; not hand sutures | |  |
|  |  |  | ○ for breast reconstruction operation with own tissue a diameter of 1.5mm is big enough | |  |
|  |  |  | ○ for the coupler system little is needed in terms of length | |  |
|  |  |  | ○ if umbilical cord has different artery wall (compared to mammary) then the coupler system should be considered | | |
|  |  |  | ○ in coupler system 2-3mm, maybe 5mm, is very easy | |  |
|  |  |  | ○ in coupler system, the vein is folded around the device | |  |
|  |  |  | ○ in the coupler system the vein must be tight, and the artery is therefore kept longer | |  |
|  |  |  | ○ measuring flow in umbilical cord | |  |
|  |  |  | ○ plastic surgeons use connectors to connect vessel in breast reconstruction operations | |  |
|  |  |  | ○ plastic surgery: 3-5mm vessels are big enough for coupler system | |  |
|  |  |  | ○ plastic surgery: mammary vein has different wall than artery | |  |
|  |  |  | ○ some sort of automatic system (click system) that connects blood vessel to blood vessel | |  |
|  |  |  | ○ the coupler mini-doppler should provide more certainty about blood flow status | |  |
|  |  |  | ○ the coupler system saves time; within 2-3 minutes completed | |  |
|  |  |  | ○ the Coupler system has different options from 1-3mm | |  |
|  |  |  | ○ the coupler system is not removed, but this has been quite a while | |  |
|  |  |  | ○ the coupler system would be possible in the umbilical cord | |  |
|  |  |  | ○ the coupler system can also have a mini doppler system that listens to the flow in blood vessel | |  |
|  |  |  | ○ the vein is very fragile, so in the coupler system is should not bend. If the artery is kept longer, the vein remains its length | | |
|  |  |  | ○ using coupler system for the artery is not successful due to wall thickness | |  |
|  |  |  | ○ with coupler flow, direct monitoring of vessels | |  |
|  |  | ● Usage of external fixation | 13 |  |  |
|  |  |  | ○ a thread around the umbilical cord can be tightened to keep it closed | |  |
|  |  |  | ○ a string around the vessel regulates bleeding when punctured | |  |
|  |  |  | ○ clip can be used on all three vessels at once or on each separately | |  |
|  |  |  | ○ H-shaped patches were used for catheter fixation | |  |
|  |  |  | ○ idea to fixation cannula on the outside | |  |
|  |  |  | ○ ideal would be to make a construction that only needs to be placed around it | |  |
|  |  |  | ○ in neonates a bridge with leucoplast is used to keep the umbilical cannula in place | |  |
|  |  |  | ○ it would be the best to clip the vessel, to cut of blood supply | |  |
|  |  |  | ○ purse string suture is used for its safety, to prevent blood loss that impairs visibility | |  |
|  |  |  | ○ some kind of device or clamp to fixate the umbilical cord | |  |
|  |  |  | ○ string around umbilical cord; with too much blood loss the string it pulled together | |  |
|  |  |  | ○ sutures outside can be done with flow is back | |  |
|  |  |  | ○ tube with extra layers is inserted in the blood vessel; a clamp is used for fixation | |  |
|  |  |  | ○ vein with navel clamp it can still bleed, but then you are prepared | |  |
| Insertion duration | Insertion duration | ● Current insertion duration | 14 |  |  |
|  |  |  | ○ 1.5-2 days after birth do not introduce anything new in umbilical vessels because neonates are colonized with bacteria | | |
|  |  |  | ○ after 1 week switch from umbilical cord to peripheral line | |  |
|  |  |  | ○ arterial line is usually up to 10 days | |  |
|  |  |  | ○ cannulation does not hold for 14-28 days | |  |
|  |  |  | ○ current umbilical vein cannulation only remains in place for a maximum of 1 week due to the risk of infection | | |
|  |  |  | ○ do not insert an umbilical cord after 24-36 hours due to increased risk of infection | |  |
|  |  |  | ○ fixation via sutures for 2 weeks | |  |
|  |  |  | ○ in 1980s in London, the arterial line was taken out after 10 days and a new one was placed | |  |
|  |  |  | ○ in radiology catheter stays in place only up to 24/48 hours | |  |
|  |  |  | ○ leave in for 2-2.5 days max (with radiology) | |  |
|  |  |  | ○ leave the umbilical artery cannulation in place for 2-3 weeks because of the high risk of infection | |  |
|  |  |  | ○ preference to remove cannulas after 1 week | |  |
|  |  |  | ○ remove cannula after one week because it can cause ischemia | |  |
|  |  |  | ○ umbilical cannula may be left in place for 1 week because of the risk of thrombosis and infection | |  |
|  |  | ● Insertion duration | 9 |  |  |
|  |  |  | ○ cannulation could remain for 4 weeks, if no infection occurs | |  |
|  |  |  | ○ do not know how long it can stay in the body | |  |
|  |  |  | ○ if cannula is inserted longer, there is an increased risk of thrombosis | |  |
|  |  |  | ○ if umbilical cannulation remains sterile, and not infection occurs, it could remain | |  |
|  |  |  | ○ permanent ascites drains, tunneled under the skin (for longer months) | |  |
|  |  |  | ○ purpose of inserting prostheses and stents for vascular surgeons is different, namely that it remains permanent | | |
|  |  |  | ○ re-cannulation in same umbilical cord vessel does not happen due to risk of sepsis and thrombosis | | |
|  |  |  | ○ replacement of the arterial line in the same vessel can cause a thrombus to move around | |  |
|  |  |  | ○ risk of inflammation/irritation after 2 days of catheter insertion | |  |
| Umbilical cord | Umbilical cord | ● Cut umbilical cord | 10 |  |  |
|  |  |  | ○ as soon as the umbilical cord is cut, and the neonate is still under water, it drowns; the oxygen supply stops | | |
|  |  |  | ○ cut the umbilical cord more in the abdomen region during caesarean to increase umbilical cord length | | |
|  |  |  | ○ do not leave umbilical stump too short, because then manipulation is no longer possible | |  |
|  |  |  | ○ in premature infants; leave 4-5cm umbilical cord to stump | |  |
|  |  |  | ○ in the current umbilical cannulation procedure in neonates it does not matter which vessel is cannulated first, it depends more on pragmatic approach | | |
|  |  |  | ○ it is possible to cut the umbilical cord more in vulva | |  |
|  |  |  | ○ sometimes premature neonates cannot be put on the stomach because the umbilical cord is too short | | |
|  |  |  | ○ where the umbilical cord is cut off depends on how the neonate is born | |  |
|  |  |  | ○ with a long umbilical cord stump, 10cm is meant | |  |
|  |  |  | ○ with caesarean possible to leave umbilical cord stump longer if preferred | |  |
|  |  | ● Dry/wet umbilical cord | 4 |  |  |
|  |  |  | ○ challenge of the experiment to keep everything dry | |  |
|  |  |  | ○ currently with normal cannulas used in neonates, air consistency does not matter | |  |
|  |  |  | ○ umbilical stump dries up during umbilical cannulation procedure | |  |
|  |  |  | ○ use sterile water and gauze around umbilical cord stump to keep moist and wet | |  |
|  |  | ● Rotations umbilical cord | 9 |  |  |
|  |  |  | ○ 1 rotation per 10cm | |  |
|  |  |  | ○ a twisted umbilical cord can be a little bit untwisted, so that the vessel is straighter | |  |
|  |  |  | ○ blood vessels revolve around each other | |  |
|  |  |  | ○ less rotations of umbilical cord in premature infants | |  |
|  |  |  | ○ less umbilical cord rotations in premature infants, due to thinner umbilical cord | |  |
|  |  |  | ○ maybe less rotation of vessel in umbilical cord in premature infants | |  |
|  |  |  | ○ research on turns per umbilical cord: how many per umbilical cord | |  |
|  |  |  | ○ rotation of umbilical cord won't be much of a problem | |  |
|  |  |  | ○ umbilical cord does not twist every few millimeters | |  |
|  |  |  | ○ when further from the abdominal skin, the umbilical cord becomes more twisted and that is more difficult | | |
|  |  | ● Substance umbilical cord | 7 |  |  |
|  |  |  | ○ more Wharton jelly may prevent vessel recognition/view | |  |
|  |  |  | ○ not easy to puncture through Wharton jelly | |  |
|  |  |  | ○ premature infants have less Wharton jelly, and thus thinner umbilical cord and more vulnerable | |  |
|  |  |  | ○ substance around the vessel is very soft | |  |
|  |  |  | ○ the jelly around the umbilical cord must be considered when cannulating | |  |
|  |  |  | ○ the umbilical cord is so soft tissue that you do not feel if you are going through a vessel | |  |
|  |  |  | ○ Wharton jelly offers little resistance | |  |
|  |  | ● Umbilical cord length | 7 |  |  |
|  |  |  | ○ devices may reduce umbilical cord length | |  |
|  |  |  | ○ do not leave umbilical stump length too long, because if a cannula is inserted all the away, it may get stuck in walls due to the windings | | |
|  |  |  | ○ in premature neonates the umbilical cord length is left longer | |  |
|  |  |  | ○ umbilical cord length at term is about 50cm | |  |
|  |  |  | ○ umbilical cord length can be longer if preferred | |  |
|  |  |  | ○ umbilical cord length in premature babies is about 25cm | |  |
|  |  |  | ○ umbilical cord length of 5 cm is achievable | |  |
|  |  |  | ○ umbilical stump often 2-3 cm | |  |
| Staff | Staff | ● Staff | 22 |  |  |
|  |  |  | ○ an extra nurse provided the material during umbilical cord cannulation | |  |
|  |  |  | ○ assistants have not often performed umbilical cord cannulation | |  |
|  |  |  | ○ at least 2 people need to help with umbilical cord cannulation procedure | |  |
|  |  |  | ○ cannulation is not possible with many people at the same time | |  |
|  |  |  | ○ cannulation with three men is too much | |  |
|  |  |  | ○ doctor assistants are also taught, so then it is 2 people | |  |
|  |  |  | ○ experienced neonatologists should puncture and cannulate | |  |
|  |  |  | ○ for PLS: two experienced people for cannulation | |  |
|  |  |  | ○ I do cannulation of umbilical cord by myself, since I am stubborn | |  |
|  |  |  | ○ in Utrecht the assistants and PAs performed cannulation | |  |
|  |  |  | ○ interventional radiologists can cannulate almost everything | |  |
|  |  |  | ○ interventional radiologists have experience with puncturing artery tibialis posterior, which is also 2mm wide | | |
|  |  |  | ○ interventions of intervention radiologist are often in children from 3-4 years | |  |
|  |  |  | ○ it may be more difficult to insert umbilical cord cannulas in peripheral hospital | |  |
|  |  |  | ○ most experienced specialist should do the cannulation | |  |
|  |  |  | ○ neonatologists are used to injecting IVs and placing lines, even in premature neonates | |  |
|  |  |  | ○ neonatologists perform umbilical cord cannula insertion | |  |
|  |  |  | ○ nurse is also present to help with materials | |  |
|  |  |  | ○ pediatric surgeons have no experience with umbilical cord, always cannulation in the neck | |  |
|  |  |  | ○ people are attached to their own way | |  |
|  |  |  | ○ people use and do certain things because their predecessor also did that | |  |
|  |  |  | ○ some PA's can canulate the umbilical cord in 5 minutes | |  |
| Anticoagulation | Coagulation | ● Alteplase | 3 |  |  |
|  |  |  | ○ alteplase is used when thrombectomy cannot be performed | |  |
|  |  |  | ○ in acute stage, alteplase can be administered locally | |  |
|  |  |  | ○ nephrologists use alteplase | |  |
|  |  | ● Cerebral hemorrhage | 3 |  |  |
|  |  |  | ○ continuous system with one vein cannula and one artery gives less risk of cerebral hemorrhages | | |
|  |  |  | ○ high risk of cerebral hemorrhages and comorbidities when using ECMO | |  |
|  |  |  | ○ premature infants have a higher risk of cerebral hemorrhages | |  |
|  |  | ● Clotting tests and times | 10 |  |  |
|  |  |  | ○ anti-X can be used to determine how much heparin is in the blood | |  |
|  |  |  | ○ anti-Xa continues to be seen as a helping value | |  |
|  |  |  | ○ anti-Xa is not a point-of-care test | |  |
|  |  |  | ○ anti-Xa is the only one that qualifies for quantitative essay and is often performed | |  |
|  |  |  | ○ anti-Xa may slightly be a more accurate measurement than the clotting test activated-clotting time | | |
|  |  |  | ○ in premature infants no shift in calcium and electrolytes is wanted | |  |
|  |  |  | ○ it is never actually known what a sufficient anti-Xa level is, even if it is measured | |  |
|  |  |  | ○ it is normal to be skeptical when it comes to coagulation measurements and its value | |  |
|  |  |  | ○ pregnant women were also examined for anti-Xa, but this required disproportionate LMWH injections, so they stopped | | |
|  |  |  | ○ the old clotting times are also no reliable anymore | |  |
|  |  | ● Coagulation ECMO/machine | 5 |  |  |
|  |  |  | ○ disadvantage of using EMCO is that a lot of anticoagulant is used | |  |
|  |  |  | ○ ECMO often has coagulation problems | |  |
|  |  |  | ○ EMCO is not used under 34 weeks due to increased risk of bleeding | |  |
|  |  |  | ○ heart-lung machine is all plastic so anticoagulant is needed | |  |
|  |  |  | ○ Radboud used anti-Xa for ECMO | |  |
|  |  | ● Coagulation general | 22 |  |  |
|  |  |  | ○ a very small amount, max 5 ml, can be given to neonate | |  |
|  |  |  | ○ before artificial womb can be used, coagulation should be in order; otherwise, same problems as ECMO | | |
|  |  |  | ○ combi poloxamers and heparin can be investigated to prevent temperature dependency, keeps it from clumping and steam | | |
|  |  |  | ○ contact system inhibitors present less bleeding risk than heparin because heparin acts centrally | |  |
|  |  |  | ○ fetal surgeons only make a small puncture in the vessels, that does not cause problem with coagulation | | |
|  |  |  | ○ if the cannula stays behind in the vessel for longer, a coating where endothelial cells can grow is preferred | | |
|  |  |  | ○ in acute bleeding Spongostan may be used, coils and again Spongostan | |  |
|  |  |  | ○ in umbilical cord cannulation the goal is not to have a perfect endothelial coating, since the cannula is also removed after a few weeks | | |
|  |  |  | ○ influencing platelet activation might also be possible for anticoagulation | |  |
|  |  |  | ○ inhibitor of contact activation also provides anticoagulant effect | |  |
|  |  |  | ○ it is good to think about interventions that inhibits the contact activation | |  |
|  |  |  | ○ less foreign material makes a difference in the extent to which contact activation is generated in the blood | | |
|  |  |  | ○ local anticoagulation is not my area of expertise, so I cannot comment on it | |  |
|  |  |  | ○ maybe something else can be given systematically, instead of heparin | |  |
|  |  |  | ○ nothing is done concerning thrombosis, just hope and pray | |  |
|  |  |  | ○ other parts of the system, besides the catheter, are reasons for systemic anticoagulation | |  |
|  |  |  | ○ prime PLS system with blood | |  |
|  |  |  | ○ Spongostan helps to stop bleeding | |  |
|  |  |  | ○ Spongostan dissolves | |  |
|  |  |  | ○ the use of citrate in heparin-free hemodialysis results in recalcification | |  |
|  |  |  | ○ vascular surgeons only use heparin, LMWH, platelet aggregation inhibitors, warfarin and other vit K antagonists in terms of coagulation | | |
|  |  |  | ○ when using whole oxygenator, you cannot escape systemic anticoagulation | |  |
|  |  | ● Coating | 25 |  |  |
|  |  |  | ○ a cannula coating is preferred that works for a while and that is not easily satisfied | |  |
|  |  |  | ○ a small amount of heparin may be released systemically via heparin coating, which is not so bad, because that is already happening | | |
|  |  |  | ○ anti-clotting coating may still be possible | |  |
|  |  |  | ○ cannula and machine coating are preferred | |  |
|  |  |  | ○ disadvantage of the coating is that the anticoagulant effect dissolves, making the catheter chemically unstable and fragmented | | |
|  |  |  | ○ disadvantage of thrombin module coating is that a lot of protein C is activated, which also increases the risk of bleeding | | |
|  |  |  | ○ do not know if coating of the entire system is reasonable and feasible due to the area and complexity | | |
|  |  |  | ○ endothelial cells on coating is not necessarily a goal for something temporary | |  |
|  |  |  | ○ example of coating is paclitaxel in stents | |  |
|  |  |  | ○ have a critical look at the release stability with coating cannulas | |  |
|  |  |  | ○ heparin-coated cannula is preferred | |  |
|  |  |  | ○ i do not know coated cannulas | |  |
|  |  |  | ○ in cardiology drug eluting stents are used; they also release some anticoagulant | |  |
|  |  |  | ○ it is also not preferred when heparin-coated release of heparin has an unpredictable course | |  |
|  |  |  | ○ it is difficult to coat a whole system/machine | |  |
|  |  |  | ○ it is good if endothelial cells settle on the catheter coating. It is less preferred that plasma protein bind to coating, as it may result in coating satisfaction. | | |
|  |  |  | ○ it would be a shame if heparin from heparin-coating comes systemically | |  |
|  |  |  | ○ other not heparin coating, but molecules coating | |  |
|  |  |  | ○ protein C could maybe act as a cannula coating | |  |
|  |  |  | ○ the manufacturers state that heparin-coating cannulas had long duration | |  |
|  |  |  | ○ the principle of heparin coating has been known for years, but has varying degrees of success | |  |
|  |  |  | ○ there is heparin coated cannulas | |  |
|  |  |  | ○ there could also be a combination of PTFE and heparin coating | |  |
|  |  |  | ○ thrombomodulin could also be used as a coating because of the anticoagulant effect | |  |
|  |  |  | ○ vascular surgeons have heparin-coated endograft | |  |
|  |  | ● Heparin | 36 |  |  |
|  |  |  | ○ a little bit of heparin is given via the line | |  |
|  |  |  | ○ a small amount of continuous heparin drip: 0.5EI per kilogram per hour | |  |
|  |  |  | ○ an extra-corpulent system that eliminates the need for heparin is preferred | |  |
|  |  |  | ○ anticoagulation can be provided by giving little heparin over a continuous intravenous line | |  |
|  |  |  | ○ despite heparin there is still occasionally thrombosis in vessels | |  |
|  |  |  | ○ despite heparin use, renal infarction due to thrombus was also seen | |  |
|  |  |  | ○ fear of intracerebral hemorrhage due to leakage of the blood-brain barrier during heparinization | |  |
|  |  |  | ○ heparin amount could work homeopathically, but not sure | |  |
|  |  |  | ○ heparin can be used based on the anti-Xa level | |  |
|  |  |  | ○ heparin dosing is difficult in neonates, soon too much is given | |  |
|  |  |  | ○ heparin has been used the most and for the longest time, so probably works best | |  |
|  |  |  | ○ heparin has not shown reduction in the risk of clots (at the catheter site) | |  |
|  |  |  | ○ heparin in stents can also dissolve | |  |
|  |  |  | ○ heparin is used in both umbilical artery and vein | |  |
|  |  |  | ○ heparin is used to keep line open and reduce risk of thrombosis | |  |
|  |  |  | ○ heparin more for treatment and alteplase is used in present clots | |  |
|  |  |  | ○ heparinization leads to cerebral hemorrhages in premature infants | |  |
|  |  |  | ○ in neonates with a closed artery, heparin bolus is given and then continuously on the pump | |  |
|  |  |  | ○ in Radboud, anti-Xa is measured as standard when heparinizing premature infants | |  |
|  |  |  | ○ in the breast reconstruction with own tissue operation, the patient is given both Fraxiparine and ascal. However, not all hospitals give ascal next to Fraxiparine | | |
|  |  |  | ○ in Utrecht continuous heparin iv is given over arterial line, not over central venous line, due to fear of thrombosis | | |
|  |  |  | ○ in Utrecht, the use of heparin was included in the protocol | |  |
|  |  |  | ○ interventional radiology uses heparin or alteplase if the vessel really has to be opened | |  |
|  |  |  | ○ intravenous NaCl works just as well as heparinization | |  |
|  |  |  | ○ it differs per hospital whether they use heparin in neonate | |  |
|  |  |  | ○ it has been investigated whether heparin could enter systemically, but I don't know exactly how that turned out | | |
|  |  |  | ○ little bit of heparin can be used, but be careful | |  |
|  |  |  | ○ look primarily at how heparin can be titrated as low as possible and control it as effectively as possible | | |
|  |  |  | ○ maybe heparin doses can be influenced by laboratory tests | |  |
|  |  |  | ○ neonatologists become restless from heparin | |  |
|  |  |  | ○ no need to be afraid of bleed at such low heparin iv dose | |  |
|  |  |  | ○ pediatric surgery: heparin is used | |  |
|  |  |  | ○ research still ends with heparin | |  |
|  |  |  | ○ some hospitals give little heparin over continuous line | |  |
|  |  |  | ○ start procedure for angioplasty in adult with bolus of 5000 units heparin | |  |
|  |  |  | ○ vascular surgeons sometimes do not even use heparin anymore | |  |
| Time frame of cannulation | Time frame of cannulation | ● Time frame of cannulation | 26 |  |  |
|  |  |  | ○ 2 minutes to cannulate | |  |
|  |  |  | ○ 2 minutes without oxygen can be acceptable | |  |
|  |  |  | ○ can give cannulation timeframe, but that is based on nothing | |  |
|  |  |  | ○ cannot say anything about duration of time that a neonate can be without oxygen | |  |
|  |  |  | ○ cannulate as fast as possible | |  |
|  |  |  | ○ cannulation can be performed within 10 minutes | |  |
|  |  |  | ○ cannulation is a matter minutes | |  |
|  |  |  | ○ cannulation must be fast | |  |
|  |  |  | ○ cannulation should be quick, but does not dare to say within what time frame | |  |
|  |  |  | ○ cannulation within 3 minutes should be possible with 2 vessels | |  |
|  |  |  | ○ experience allows for faster cannulation time | |  |
|  |  |  | ○ if all goes well, all cannulas can be installed within 5 minutes, but sometimes it takes an hour | |  |
|  |  |  | ○ in breech delivery it takes 2-3 minutes before damage occurs | |  |
|  |  |  | ○ in PLS: cannulation is performed under time pressure | |  |
|  |  |  | ○ in situations outside the abdomen, sometimes nothing happens in the first two minutes | |  |
|  |  |  | ○ investigate time without oxygen with digital-twin research | |  |
|  |  |  | ○ neonate could go without blood supply for 1-2 min | |  |
|  |  |  | ○ neonate in breech position must be born within 3 minutes | |  |
|  |  |  | ○ not much time for cannulation | |  |
|  |  |  | ○ one method is easier but costs less time, the other method is more complex, but you have more time | | |
|  |  |  | ○ only for a very short time no cannula in artery (when there is one in the vein) | |  |
|  |  |  | ○ preferred: to interrupt blood flow for as short as possible | |  |
|  |  |  | ○ puncturing vein should be possible within 2 minutes (also in the event of engorgement), arteries would be a challenge because there are 2 vessel | | |
|  |  |  | ○ the cannulas should be inserted if placenta functions, which is probably a few minutes | |  |
|  |  |  | ○ the umbilical cord line procedure easily takes 10 minutes, more often 20 minutes | |  |
|  |  |  | ○ umbilical vein cannulation is done within 1 minute | |  |
| Future experiments | Future experiments | ● Experiments | 12 |  |  |
|  |  |  | ○ advice type article about glue | |  |
|  |  |  | ○ cannulation method trial and error | |  |
|  |  |  | ○ develop and test theory in models | |  |
|  |  |  | ○ experiment to see if superglue stays on | |  |
|  |  |  | ○ experiment with a device that fixate vessel before cannulation | |  |
|  |  |  | ○ experiment with super glue | |  |
|  |  |  | ○ experiment may look at perfusion procedures | |  |
|  |  |  | ○ how to fix cannula; maybe via glue | |  |
|  |  |  | ○ make an artificial umbilical cord with 3 small catheters, which you must slide and glue on, while watching them stay on | | |
|  |  |  | ○ need a catheter for experimenting with glue | |  |
|  |  |  | ○ testing coating material in animals or in vitro experiments | |  |
|  |  |  | ○ vascular surgeon advised contacting GORE, from gore | |  |
|  |  |  | ○ years ago, the Gore company made a stent that can be inserted into the blood from a distance | |  |
| Vasospasm | Vasospasm | ● Lidocaine | 8 |  |  |
|  |  |  | ○ during the entire operation vasospasm was a problem, however, a little bit of lidocaine helps | |  |
|  |  |  | ○ give lidocaine against vasoconstriction of the umbilical cord arteries | |  |
|  |  |  | ○ in Utrecht no lidocaine is used | |  |
|  |  |  | ○ lidocaine was dripped onto vessel | |  |
|  |  |  | ○ lidocaine was never used | |  |
|  |  |  | ○ unsure if lidocaine really works; just advice from predecessors | |  |
|  |  | ● Papaverine | 5 |  |  |
|  |  |  | ○ ideally, do not use papaverine | |  |
|  |  |  | ○ in the current neonatal setting: the neonate does not depend on the umbilical cord that supply oxygen and blood flow. This differs in PLS setting. | | |
|  |  |  | ○ in the current setting, giving papaverine is avoided | |  |
|  |  |  | ○ papaverine is used in lamb experiments to prevent vasospasm | |  |
|  |  |  | ○ something papaverine is only used for short period of time | |  |
|  |  | ● Vasospasm general | 21 |  |  |
|  |  |  | ○ adults only get minimal dose of vasospasm | |  |
|  |  |  | ○ assumption that warm water reduces vasospasm | |  |
|  |  |  | ○ cardiologists also use vasospasm cocktail (via wrist) | |  |
|  |  |  | ○ cocktail to prevent spasm: verapamil and nitroglycerines | |  |
|  |  |  | ○ cutting the umbilical cord causes it to squeeze | |  |
|  |  |  | ○ do not give vasoconstrictor substances | |  |
|  |  |  | ○ do not know how arteries respond in terms of vasospasm to the 2 puncture techniques | |  |
|  |  |  | ○ in ECMO lidocaine drops are often used to counteract vasospasm | |  |
|  |  |  | ○ ischemia must recover quickly after catheter insertion, otherwise catheter out | |  |
|  |  |  | ○ no specific actions to counteract vasospasm | |  |
|  |  |  | ○ prevent squeezing as it causes vasoconstriction and vessel damage | |  |
|  |  |  | ○ probably necessary to give something to prevent vasospasm | |  |
|  |  |  | ○ puncturing x number of centimeters before the abdominal inserting does not cause spasm | |  |
|  |  |  | ○ the goal is to ensure that no constriction of the umbilical cord occurs | |  |
|  |  |  | ○ unclear how long it takes for vasospasm to occur and how long it lasts | |  |
|  |  |  | ○ vasospasm cocktail may vary in doses | |  |
|  |  |  | ○ vasospasm gives no problem in children with spleen embolization and varicocele surgery | |  |
|  |  |  | ○ vasospasm is not a problem because you do not cut through it | |  |
|  |  |  | ○ vasospasm is only temporary | |  |
|  |  |  | ○ when the artery is excited, a spasm quickly occurs and then you cannot enter it | |  |
|  |  |  | ○ with coupler system there is no increased risk of vasoconstriction | |  |

**Appendix B -** Coding tree focus groups

| **Main category** | **Groups** | **Code** | **Groundedness** |
| --- | --- | --- | --- |
| Cannula fixation | The usage of stent | ● Advantage of stent | 3 |
|  |  |  | ○ self-expendable stents dilate with vessel |
|  |  |  | ○ stent is somewhat larger, can be in vessel wall, rapid fixation and may grow with vessel |
|  |  |  | ○ stents are useful at first because of rapid canalization, inflation, and fixation |
|  |  | ● Side effect stent | 1 |
|  |  |  | ○ disadvantage of self-expandable stents is piercing in the vessel wall; it could react to this, and it takes up space in the vessel |
|  | The usage of sutures | ● Advantage of sutures | 1 |
|  |  |  | ○ sutures do not stretch with vessel growth |
|  |  | ● Side effects of suturing | 1 |
|  |  |  | ○ in the Australian group the sutures came loose, and the sheep bled |
|  |  | ● Current usage sutures | 5 |
|  |  |  | ○ a hemostatic suture is relatively tight around the cannula |
|  |  |  | ○ Flake uses microsurgical technique to suture according to end-to-end |
|  |  |  | ○ Flake's microsurgery ensures that it fits together well |
|  |  |  | ○ maybe combining glue and sutures is necessary |
|  |  |  | ○ vascular surgeon would bet money on suturing and play with glue |
|  | The usage of glue | ● Side effect of glue | 4 |
|  |  |  | ○ glue is used for surface and to stop bleeding, not for fixation |
|  |  |  | ○ glue is used for the surface, not intra-abdominal |
|  |  |  | ○ it is thought that toxicity of glue is safe |
|  |  |  | ○ vascular surgeons have no experience with glue |
|  |  | ● Advantage of glue | 0 |
|  |  |  |  |
|  |  |  |  |
|  | Other fixation options | ● Extern fixation | 14 |
|  |  |  | ○ balloon can put pressure on the vessel wall, and why is it necessary if it can be fixed on the outside |
|  |  |  | ○ enough time for fixation when fixating on the outside |
|  |  |  | ○ for fixation suture can be placed around vessel and then tightened |
|  |  |  | ○ ideally one thread around the umbilical cord |
|  |  |  | ○ maybe put some super glue on the outside |
|  |  |  | ○ mid-cut option and fixation of vessel with proximal and distal temporally clamp for cannula fixation |
|  |  |  | ○ one wire around the entire umbilical cord is insufficient for three catheters |
|  |  |  | ○ preference is fixation on the outside of the cannula |
|  |  |  | ○ preference is for sutures on the outside that ensures that the vessel remains in contact with the cannula |
|  |  |  | ○ proximally and distally vessel reined to cut halve circumference in the middle |
|  |  |  | ○ string around the cannula can solve the problem of tight closure of the cannula and blood vessel |
|  |  |  | ○ there could more space at the tip of the cannula, but then you have to place the suture closer to the cannula tip |
|  |  |  | ○ vessel can be reined |
|  |  |  | ○ with cannula used, slide tube into vessel, easy fixation (via outside wire or glue) |
|  |  | ● Coupler system | 2 |
|  |  |  | ○ end-to-end device from plastic surgery is very attractive, even without any previous experience |
|  |  |  | ○ maybe Coupler system used |
| Physiology | Physiology | ● Amniotic fluid | 2 |
|  |  |  | ○ an option is to often change amniotic fluid, so no antibiotics need to be used, or put antibiotics in amniotic fluid |
|  |  |  | ○ if the system works well, we hope to prevent infection by changing/renewing amniotic fluid |
|  |  | ● Antibiotics | 7 |
|  |  |  | ○ in ideal situation, no antibiotics |
|  |  |  | ○ in lamb research many antibiotics are used |
|  |  |  | ○ neonates are also given antibiotics |
|  |  |  | ○ nowadays antibiotic is almost always given if mother develops a fever during childbirth |
|  |  |  | ○ premature neonates develop NEC more often when wide-spectrum antibiotic is used |
|  |  |  | ○ The disadvantage of antibiotics is that the intestinal flora of neonates is affected |
|  |  |  | ○ using more specific antibiotics, less NECs is developed in premature neonates |
|  |  | ● Brain | 1 |
|  |  |  | ○ sheep brain developments faster in which brain function matures earlier |
|  |  | ● Circulation and pressure | 2 |
|  |  |  | ○ preterm infants are still completely autonomic dysregulated |
|  |  |  | ○ with fetal circulation and the shunts less pressure differences occur |
|  |  | ● Intestinal flora | 3 |
|  |  |  | ○ fetuses have no intestinal flora |
|  |  |  | ○ intestines are just as premature as lungs, so an option is nutrition via umbilical cord |
|  |  |  | ○ neonates can develop NEC from food for which their intestines are not yet ready |
|  |  | ● Ischemia | 3 |
|  |  |  | ○ discoloration is immediately visible in most umbilical artery line |
|  |  |  | ○ how can the skin color be monitored under water |
|  |  |  | ○ removing intravenous line often solves the ischemic problem |
|  |  | ● Resistance | 1 |
|  |  |  | ○ resistance must be low; so larger diameter results is less resistance |
| Canulation technique | Insertion via complete/transversal dissection | ● Advantage of complete dissection | 5 |
|  |  |  | ○ end-to-end technique provides faster vessel identification |
|  |  |  | ○ Flake did end-to-end |
|  |  |  | ○ preference is complete cutting through (dissection) for vessel recognition and to not damage intima/endothelium |
|  |  |  | ○ with end-to-end little chance of cutting through vessel |
|  |  |  | ○ with Flake: end-to-end suturing was fast, within a few minutes |
|  |  | ● Side effects of transversal dissection | 4 |
|  |  |  | ○ considerable time pressure when using ed-to-end |
|  |  |  | ○ when doing complete dissection, the vessel shoots into Wharton's jelly and then the vessel is lost |
|  |  |  | ○ with cutting though (complete dissection) control over vessel wall in difficult |
|  |  |  | ○ with end-to-end complete ischemia is left |
|  | Insert via side-entry | ● Advantage of partial incision | 9 |
|  |  |  | ○ after partial incision immediately insert the cannula, without first inserting the needle |
|  |  |  | ○ easier to pull umbilical cord over finger for traction |
|  |  |  | ○ in port-a-cath for chemotherapy is side-entry also used |
|  |  |  | ○ not completely cut through but large enough to insert cannula |
|  |  |  | ○ the advantage of partial cutting is that the vessel is less frequent lost/shoots away |
|  |  |  | ○ the advantage of partial transection is that it maintains circulation and not suddenly lose a vessel |
|  |  |  | ○ when inserting in the side, no spasm will develop |
|  |  |  | ○ with just a little cut the vessel might not shoot away |
|  |  |  | ○ with partial incision, vessel length remains intact |
|  |  | ● Advantage of Seldinger technique | 2 |
|  |  |  | ○ Seldinger technique is used within the neonatology when using thorax drains and long lines via iv |
|  |  |  | ○ theoretical in favor of Seldinger-like technique compared to complete dissection |
|  |  | ● Side effect of partial incision | 0 |
|  |  | ● Side effect Seldinger | 7 |
|  |  |  | ○ and damaged |
|  |  |  | ○ not clear whether Seldinger will work with Wharton jelly |
|  |  |  | ○ Seldinger is difficult with small vessel |
|  |  |  | ○ with Seldinger technique it is difficult to navigate through Wharton's jelly, to enter the vessel |
|  |  |  | ○ with Seldinger technique more damage than cutting through completely |
|  |  |  | ○ with Seldinger technique there is a high risk of vascular damage |
|  |  |  | ○ with Seldinger there is a chance of spasm, which increases its difficulty of its incision |
| Cannulate | Cannulate | ● Depth of cannula insertion | 9 |
|  |  |  | ○ Inserting cannula 1-2 cm at 10 cm from the abdomen proved to be the best result |
|  |  |  | ○ cannulating a small piece in the umbilical cord may have a kind of homeostatic effect that may keep it working |
|  |  |  | ○ every time cannula is replaced, less centimeters of the umbilical cord are left |
|  |  |  | ○ Flake keeps 10 cm between cannulation and abdomen, but why not 20 cm? |
|  |  |  | ○ in sheep experiments the first catheter very placed deep because they thought it would fixed well, but no good results were found |
|  |  |  | ○ not the intention to insert a catheter into the abdomen, plus then also less chance of leg ischemia |
|  |  |  | ○ one wire around all 3 vessels is optional but determined by how far catheter is advanced |
|  |  |  | ○ there is a risk that if you put in a little bit, the chance that it will come out is greater |
|  |  |  | ○ when advancing the catheter briefly, it is important that you first fix the vessel before moving on to the next catheter |
|  |  | ● Dilatation | 3 |
|  |  |  | ○ a port-a-cath set also has a small hook that can be used to pick up the vessel wall, to insert the cannula easier |
|  |  |  | ○ hook can be used to hold vessel open for better access |
|  |  |  | ○ maybe make the diameter of the cannula larger than the vessel, only then the rest of the vessel will still be small |
|  |  | ● Cannulation requirement | 9 |
|  |  |  | ○ bigger problem with growing neonate and 1 cannula is that you want to achieve more flow as the neonate grows |
|  |  |  | ○ cannula may need to be replaced due to the growth of the baby |
|  |  |  | ○ cannulation practice on model |
|  |  |  | ○ ECMO forbitten under 34 weeks due to increased risk of bleeding |
|  |  |  | ○ ECMO must provide good flow |
|  |  |  | ○ important to have sufficient umbilical cord length |
|  |  |  | ○ large cannulas are preferred to get enough flow over ECMO |
|  |  |  | ○ possible problem: replacing cannula as vessel enlarges |
|  |  |  | ○ think that cannula will probably be too small instead of too big. |
|  |  | ● Recognizing vessel | 5 |
|  |  |  | ○ color doppler is very easy to visualize vessels |
|  |  |  | ○ echo use beforehand to identify vessels |
|  |  |  | ○ identifying vessels when cutting is not possible within 2, often not within 5-10 minutes |
|  |  |  | ○ make vessel more visible with ultrasound, but how to use it in cannulation is difficult |
|  |  |  | ○ no experience with the technique of ultrasound identification by a vascular surgeon |
| Insertion duration cannula | Insertion duration | ● Insertion duration | 1 |
|  |  |  | ○ cannula inserted for 4 weeks, then high risk of thromboembolic events |
| Umbilical cord | Umbilical cord | ● Substance umbilical cord | 2 |
|  |  |  | ○ umbilical cord has special anatomy because you have to work in Wharton's jelly with a contracted artery. There is hardly any grip |
|  |  |  | ○ worried about catheter falling out if only inserted a little bit |
|  |  | ● Size vessels | 3 |
|  |  |  | ○ maybe with artery 0.4-0.8mm |
|  |  |  | ○ umbilical cord vessels 2-3mm |
|  |  |  | ○ with artery count on 1-1.5mm |
| Staff | Staff | ● Staff | 2 |
|  |  |  | ○ neonatologists are involved in supervising the neonate and they already have the skill cannulation |
|  |  |  | ○ vascular surgeons are no longer asked for cannulation |
| Anticoagulation | Coagulation | ● Alteplase | 1 |
|  |  |  | ○ example: a term neonate lost a kidney but still didn't use alteplase because there are 2 kidneys and only 1 brain |
|  |  | ● Clotting values | 2 |
|  |  |  | ○ do not make decisions based on PT or anti-Xa levels |
|  |  |  | ○ it is not possible to monitor blood values and adjust clotting accordingly |
|  |  | ● Coagulation general | 3 |
|  |  |  | ○ coagulation should act locally, avoid systemically |
|  |  |  | ○ preterm infants have impaired coagulation, prolonged clotting times and increased risk of cerebral hemorrhage |
|  |  |  | ○ studies on coagulation contain percutaneous lines, not umbilical lines |
|  |  | ● Coating | 8 |
|  |  |  | ○ if you do not want to give heparin systematically, it makes sense to work with heparin-coated material |
|  |  |  | ○ in theory, coating would be sufficient in terms of anticoagulation |
|  |  |  | ○ it is less likely that coated heparin will dissolve and come systemically |
|  |  |  | ○ neonatologists do not know any cannulas that are heparin coated that are used, but there are vascular prostheses |
|  |  |  | ○ oxygenator must also be coated or with alternatively |
|  |  |  | ○ there is doubt whether coating works for 4 weeks, but manufacturers claim that they work for a long time, at least 4 weeks |
|  |  |  | ○ wall can also be coated for anti-clotting |
|  |  |  | ○ with vascular prostheses they claim that heparin remains on the wall of the prostheses for a long time |
|  |  | ● Heparin | 6 |
|  |  |  | ○ 5-5 division center in NL uses heparin |
|  |  |  | ○ extracorporeal machines always require heparin |
|  |  |  | ○ someone with alternatives to heparin |
|  |  |  | ○ in studies with lambs, low doses of heparin were used |
|  |  |  | ○ not much evidence for heparin, often through training from predecessor |
|  |  |  | ○ profit of heparin is minimal in central venous lines on thromboembolic events and infection |
|  |  |  | ○ studies evidence for heparin vs saline are not very strong |
|  |  | ● Stroke | 4 |
|  |  |  | ○ Flake thinks neonatal cerebral hemorrhages could be from ventilators, the pressure and pressure differentials getting it premature |
|  |  |  | ○ keeping fetus in fetal circulation for longer time could maybe decrease the risk of hemorrhage |
|  |  |  | ○ little vasoactive medication already causes cerebral hemorrhage |
|  |  |  | ○ no cerebral hemorrhages were seen in sheep |
| Vasospasm | Vasospasm | ● Lidocaine | 9 |
|  |  |  | ○ caution with lidocaine |
|  |  |  | ○ heart block is blockade of sinus node by lidocaine |
|  |  |  | ○ in Radboud lidocaine drops were used |
|  |  |  | ○ lidocaine could cause heart block if it comes systemically |
|  |  |  | ○ lidocaine first causes excitation/spasm and in a later phase dilation |
|  |  |  | ○ lidocaine ointment is already not smeared on the neonates' back due to possible systemic effects |
|  |  |  | ○ lidocaine was not used in 4 center |
|  |  |  | ○ the question is whether lidocaine really works, because it came across something that was given down by predecessor |
|  |  |  | ○ vasospasm with lidocaine can last 2 min |
|  |  | ● Nitroglycerin | 12 |
|  |  |  | ○ gastroenterology use nitrate ointment for fissure ani |
|  |  |  | ○ gyneacologists has no experience with nitroglycerin |
|  |  |  | ○ the catheter had already been removed, then you tried to restore the circulation with nitroglycerin |
|  |  |  | ○ low dose of nitroglycerin in system can have big consequences for little ones |
|  |  |  | ○ nitrates work on smooth muscles tissues |
|  |  |  | ○ nitroglycerin can be given under the tongue |
|  |  |  | ○ nitroglycerin may also work on the umbilical cord if it works as an ointment |
|  |  |  | ○ nitroglycerin plasters were used if there was no adequate coloration |
|  |  |  | ○ nitroglycerine is used for relaxation of the uterus |
|  |  |  | ○ unclear if nitroglycerin works on umbilical cord |
|  |  |  | ○ vascular surgeon could place the umbilical cord in a nitroglycerin bath |
|  |  |  | ○ vascular surgeons have no experience with nitroglycerin |
|  |  | ● Papaverine | 10 |
|  |  |  | ○ flake used papaverine, enthusiastic about it |
|  |  |  | ○ if papaverine is not inject into the vessel, it may have less effect on the neonate |
|  |  |  | ○ maybe inject papaverine into Whartons jelly |
|  |  |  | ○ papaverine in Whartons jelly give mechanical pressure |
|  |  |  | ○ papaverine is injected in tissue around the vessel |
|  |  |  | ○ papaverine is often used in shunt-surgery (according to vascular surgeon) |
|  |  |  | ○ papaverine is put into vessel without going into circulation, and over the vessel |
|  |  |  | ○ papaverine works continues differently, but does work |
|  |  |  | ○ unclear whether Flake randomized usage of papaverine, or if he looked at other possibilities |
|  |  |  | ○ when papaverine used as a prep, then put the umbilical cord in it |
|  |  | ● Vasospasm general | 6 |
|  |  |  | ○ in our situation of fast cannulation, dilatation is not desirable in later phase |
|  |  |  | ○ lidocaine, papaverine and nitroglycerine for vasospasm have emerged |
|  |  |  | ○ mechanical manipulation by injecting vasodilation fluid can get mechanically stuck |
|  |  |  | ○ takes time for vessels in vasospasm to open to access |
|  |  |  | ○ umbilical cord insertion causes vasospasm |
|  |  |  | ○ vasospasm is big problem |
